# Application of Paper-Based Microfluidic Analytical Devices (µPAD) in Forensic and Clinical Toxicology: A Review

**DOI:** 10.3390/bios13070743

**Published:** 2023-07-18

**Authors:** Giacomo Musile, Cristian Grazioli, Stefano Fornasaro, Nicolò Dossi, Elio Franco De Palo, Franco Tagliaro, Federica Bortolotti

**Affiliations:** 1 Unit of Forensic Medicine, Department of Diagnostics and Public Health, University of Verona, P.le Scuro 10, 37134 Verona, Italy; 2 Department of Agrifood, Environmental and Animal Science, University of Udine, Via Cotonificio 108, 33100 Udine, Italy; 3 Department of Chemical and Pharmaceutical Sciences, University of Trieste, Via L. Giorgeri 1, 34127 Trieste, Italy; 4 Laboratory of Pharmacokinetics and Metabolomics Analysis, Institute of Translational Medicine and Biotechnology, I.M. Sechenov First Moscow State Medical University, Bolshaya Pirogovskaya Street, 119991 Moscow, Russia

**Keywords:** paper-based devices, forensic toxicology, clinical toxicology, point-of-need (PON) devices, microfluidics, illicit drugs, drugs, drug-facilitated sexual assault (DFSA), drug-facilitated crimes (DFCs), toxicant, biosensor

## Abstract

The need for providing rapid and, possibly, on-the-spot analytical results in the case of intoxication has prompted researchers to develop rapid, sensitive, and cost-effective methods and analytical devices suitable for use in nonspecialized laboratories and at the point of need (PON). In recent years, the technology of paper-based microfluidic analytical devices (μPADs) has undergone rapid development and now provides a feasible, low-cost alternative to traditional rapid tests for detecting harmful compounds. In fact, µPADs have been developed to detect toxic molecules (arsenic, cyanide, ethanol, and nitrite), drugs, and drugs of abuse (benzodiazepines, cathinones, cocaine, fentanyl, ketamine, MDMA, morphine, synthetic cannabinoids, tetrahydrocannabinol, and xylazine), and also psychoactive substances used for drug-facilitated crimes (flunitrazepam, gamma-hydroxybutyric acid (GHB), ketamine, metamizole, midazolam, and scopolamine). The present report critically evaluates the recent developments in paper-based devices, particularly in detection methods, and how these new analytical tools have been tested in forensic and clinical toxicology, also including future perspectives on their application, such as multisensing paper-based devices, microfluidic paper-based separation, and wearable paper-based sensors.

## 1. Introduction

Even if poisons and poisoning date back to the dawn of civilization, only in the first decades of the sixteenth century did the alchemists propose the grounds of modern toxicology, which is inspired by the concept that each compound is a poison, and there is nothing which is not: only doses eliminate the toxic effect. In fact, the famous Paracelsus affirmed: “*Omnia venenum sunt: nec sine veneno quicquam existit. Dosis sola facit, ut venenum non fit*” [1].

Considering Paracelsus’s statement, thousands of substances could hypothetically act as harmful agents. However, geographic areas and different contexts are the main factors that influence the frequency of intoxications caused by a compound. Notwithstanding this, the sectors where toxicological analyses are mostly required include a list of seven categories: (i) clinical and forensic identification of poisoning and intoxication; (ii) workplace drug testing and occupational toxicology; (iii) in utero drug exposure and exposure to drugs in childhood; (iv) drug-related unfitness to have child custody; (v) driving under the influence of drugs; (vi) patient monitoring treatment programs; and (vii) doping control [2,3].

The contexts and purposes of analytical determinations differ greatly in these different categories and, consequently, in each category, the choice of the analytes of interest, the needed sensitivity and specificity of the analytical methods, the analytical costs, and the costs of the human resources required must be chosen with the highest consciousness.

In the first category, the target analytes are the parent drugs and/or their active metabolites in order to correlate a clinical condition or death to the intake of a specific compound. Of course, in a clinical context, the time from the diagnostic suspicion to reporting a result is crucial, and any delay could be harmful for the patient. Consequently, it is of the highest importance that an analytical screening is carried out as soon as possible. It also must be considered if the clinic where the patient is treated is not fully equipped for toxicological assays.

Occupational medicine includes different areas of toxicological interest spanning from the certification of fitness-to-work (particularly in the case of safety-sensitive jobs) to the certification of an occupational disease caused by exposure to industrial toxicants. In the first instance, a preliminary assessment of the toxicological condition is often required at the workplace, while in the case of a positive result, a confirmatory test can be carried out later in a certified laboratory. Of course, the toxicological investigation of an occupational disease does not need urgent analysis and requires the analysis of multiple biological samples and often a full metabolite panel of the toxicants of interest.

Toxicological monitoring during pregnancy is performed to avoid fetal exposure to psychoactive substances and xenobiotics which could be harmful to the fetus. It has been largely demonstrated that in utero exposure to cocaine and amphetamine can cause complications during pregnancy and increase the probability of premature birth, while intellectual disability is associated with benzodiazepine abuse [2]. Drug testing in newborns is required when withdrawal syndrome is suspected because of the mother’s drug abuse history [2]. For this purpose, the biological matrices both of the newborn and the mother are analyzed; on the other hand, the father and siblings are also monitored to evaluate the fitness of the family environment to have custody of the child. All these procedures would benefit from the possibility of carrying out the analysis without the delays imposed by specimen delivery.

The evaluation of an altered psychophysical state while driving is a fundamental piece of evidence in any investigation concerning highway safety, traffic crashes, and any other vehicle collisions. Although the legal consequences of a toxicological assay require the use of the most sensitive and specific technologies, the investigation would profit highly from the availability of a rapid toxicological screening.

Drug monitoring therapy, including programs for drug abuse dependence, and doping controls are generally based on tests performed offsite, with delayed results. In fact, the scope of substance testing in drug monitoring therapies is aimed at evaluating patient compliance with the treatment and, in the case of drugs with a narrow therapeutic index, for optimizing the drug dosage. In the second case, the quantitative determination of molecules improving athletic performance is performed to avoid fraud in sports and to protect athletes’ health. Also, in the last two contexts, even if a formal report must originate from nonequivocal identifications using sophisticated instrumentation, the availability of rapid, onsite toxicological information would be greatly beneficial.

From an analytical point of view, given the very different natures and structures of the toxicants to be investigated and the different purposes of the assay, broad-spectrum detection and identification are needed, requiring the integration of multiple analytical strategies. A typical framing of this approach includes the adoption of analytical techniques which are classified into two categories: “screening” and “confirmation” assays. The difference depends on the type of information which can be obtained. The former category, essentially, consists of immunoassays and color tests, while the second one is mainly represented by methods using chromatography and mass spectrometry. The integration of the two analytical steps varies according to the different contexts and purposes in which the toxicological investigation is carried out, but in general, the two steps are applied sequentially in order to maximize diagnostic sensitivity and specificity (*Sensitivity = [TP/(TP + FN)] × 100 Specificity = [TN/(TN + FP)] × 100*, where TP stands for true positive, FN stands for false negative, TN stands for true negative, and FP stands for false positive). In particular, screening tests are chosen in order to identify all the samples containing the analyte, even accepting a number of samples falsely identified as containing the analyte (false positives). The confirmatory test is aimed at excluding false positives from the preliminary results of the screening test. On the quantification side, only confirmation techniques are believed to be suitable for this purpose. Although this approach represents a widely accepted strategy in analytical toxicology, it shows some points of weakness such as the long turn-around time, the high analytical costs, and, above all, the impossibility of performing the test at the point of need (PON). These limits are particularly relevant in specific contexts, such as emergency medicine, and in general when rapid result reporting is needed and in low-resources environments.

In recent decades, several research groups have proposed detection strategies that would allow for measurements at the PON, proposing the use of portable instruments based on different detection strategies. In fact, recent technological improvements have allowed for the miniaturization of large-size equipment, such as mass spectrometers [4] and Raman [5] and near-infrared [6] spectrophotometers. However, these advanced techniques are still too expensive and require expert handling. Also, although portable electrochemical sensors are cheaper than miniaturized spectrometers and spectrophotometers, the conventional electrode configuration may result in the equipment being difficult to handle and not suitable for practical uses [7]. On the contrary, the use of microfluidics in capillaries or chips is widely recognized as one the most promising technologies in terms of affordability, portability, and ease of use. However, the enlargement of the panel of analytes and the needed increase in sensitivity are associated with the reduced portability of devices that require pumps, valves, and complex electronics [8].

Alternatively, several research groups have proposed an alternative to sophisticated microfluidic devices: the use of paper as an inert analytical support. In fact, the capillarity of paper allows the solution to flow without external tools, thus limiting the costs of the more sophisticated microfluidic devices. Already in 1949, Müller and Clegg first reported the production of a paper device by melting paraffin on filter paper to create a channel delimited by hydrophobic paraffin barriers. The shape of the channel was intended to limit the lateral spread of the chromatographic bands typical of paper chromatography [9]. Unfortunately, the success of this original idea was delayed for almost 60 years. In fact, only in 2007, Whitesides’ research group re-proposed paper as a support for producing diagnostic devices, using photolithography to produce hydrophobic barriers [10]. Since this report, the use of paper-based analytical devices has been proposed for applications in different analytical fields, such as environmental analysis [11], personalized healthcare diagnostics [12], forensics [13], food adulteration [14], and pharmaceutical analysis [15]. This is witnessed by over 1800 scientific papers, which can be retrieved from Scopus using the following keywords: paper-based microfluidic devices (accessed on 15 March 2023). It is worth noting that paper-based technology proved to meet most of the WHO’s “RE-ASSURED” criteria (real-time analysis, easy sample collection, affordable, sensitive, selective, user friendly, and deliverable to the end user) [16]. Although these criteria have been developed for PON devices applied to clinical diagnostics, investigative actions related to the legal fields (e.g., abuse of pharmaceuticals, use of drugs of abuse, cases of poisoning and intoxication, workplace drug testing, doping control, exposure to drugs in childhood, and assessment of drug use in child custody cases) could also benefit enormously from their implementation in forensic science. In particular, the technology has been proposed for analyzing DNA [17], estimating the time since death [18], detecting explosives [19], performing serological investigation [20], and assessing urine tampering [21]. Moreover, paper micro-devices have also been used to clarify the composition of seized materials and looking for contents of illicit drug [22] and cutting agents [23].

The present report is a critical review of procedures based on µPAD technology for detecting drugs and toxicants in biological fluids (Table 1). While the first part is focused on the main technological aspects and on detection methods, i.e., colorimetry, electrochemical, and spectroscopy (Raman-based techniques and fluorescence), the second part is devoted to application methods. A final section describes the future of this technology as an almost ideal tool for PON analysis.

## 2. Paper-Based Analytical Devices

The design of paper-based devices focuses on three main aspects: (i) support; (ii) fabrication method; (iii) detection strategy.

### 2.1. Selection of Paper

The selection of the most suitable paper support is a function of porosity, thickness, price, and absorption capability. At the moment, filter paper and chromatography paper are the most used [8,28]; however, printing and office paper have also been proposed for fabricating paper-based analytical devices [29]. However, since the latter supports are treated with mineral fillers, such as calcium carbonate, to improve the paper’s properties (e.g., porosity, light scattering, gloss, and printability) their addition may interfere with some applications [30]. However, it has to be pointed out that in the fabrication of electrochemical paper-based devices (ePADs), the presence of fillers is needed to grant electric conductivity (carbon-based electrodes) [31]. In addition, other materials have been used such as nitrocellulose and fiber-based materials, including polytetrafluoroethylene (PTFE), glass fibers, graphene and graphene oxide, polypropylene, poly(lactic acid), and carbon nanotubes. In fact, because of the nitro groups, the use of nitrocellulose has provided strong noncovalent interactions with biomolecules [32], while the latter class of supports provides mechanical strength, surface hydrophobicity, porosity, and reactivity for the modification of the surface.

### 2.2. Fabrication Procedures

In addition to photolithography [10], first used in 2007, different strategies for fabricating paper-based devices have been proposed. The problems related to the rigidity of the paper support, manufacturing cost, and the multistep procedure have stimulated several researchers to propose devices that are more flexible, affordable, and easy to fabricate. A brief explanation of the methods proposed after the photolithography method is reported below.

Photolithography. Photolithography was the first patterning method for the production of paper-based analytical devices [10]. Photolithography is based on the use of a covering mask on paper impregnated with a light-sensitive solution. Although the approach enables high-resolution channels to be obtained, the entire process is expensive.

Plasma Treatment. Alternatively, Li et al. [33] first proposed plasma-based treatment to make the paper support hydrophobic via the addition of alkyl ketene dimer and using metal masks. Although plasma treatment has fewer steps than photolithography, expensive plasma oxidizers and a mask are required to generate barriers.

Wax Printing. In 2009, Lu et al. [34] and Carrilho et al. [35] independently proposed the use of wax printers to create paper-based analytical devices. After designing and printing the pattern of the device on the paper, the hydrophobic barriers are created by melting wax through the paper. This fabrication method has some advantages, such as affordability, simplicity, rapidity, and robustness; however, the resolution is low, and the wax barriers are not compatible with most organic solvents. While wax printing is attractive, the commercial availability of wax printers has been discontinued. In addition to the wax printing method, 3D wax printing with a custom-made extruder was used to print patterns with a high resolution, obtaining a higher resolution than that obtained with the common 2D wax printers [36]. Also, Le et al. [37] proposed a CO_2_ laser method consisting of a single-step approach, including the heating, melting, penetration, and solidification of the wax.

Flexography, Inkjet, and Laser Printing. Alternative printing methods have been based on flexography, ink-jetting, and toner-based printing. In the flexographic method, polystyrene in an organic solvent (i.e., toluene or xylene) is used as printing ink on a roll to form hydrophobic barriers not requiring heat treatment [38]. In 2008, Abe et al. [39] proposed a strategy for fabricating a µPAD using inkjet printing not only for creating hydrophobic barriers but also for depositing reagents. In particular, the inkjet was used to locally dissolve a preformed hydrophobic poly(styrene) layer, and a cartridge filled with reagents was then used for depositing the reagents. The inkjet printing approach has been used to deposit hydrophobic acrylate ink, and a UV light source is used to cure the ink [40]. Wang et al. alternatively proposed a sol-gel method based on acid-hydrolyzed methyltrimethoxysilane (MTMS) used as ink [41]. Toner-based printing systems have also been proposed for manufacturing paper-based devices, and as per wax printing, a heating step follows the printing phase; however, the temperature required for melting the toner is higher, causing the modification of the paper structure [42].

Pen writing. This method is based on creating hydrophobic channels on paper during the evaporation of the solvent from the pen. Different kinds of pens have been used, such as permanent marker pens [43], eyeliner pencils [44], and 3D pens [45]. Automatizing the system by means of a plotter permitted the improvement of the reproducibility and resolution [46].

Wax dipping and glue spraying. In these techniques, a magnetic mask is used to protect the hydrophilic portion of the paper which will be used for channels. The assembly was submerged into melted wax [47] or sprayed with scholar glue [48]. For the former, the assembly is cooled, and the magnets were then removed, leaving hydrophilic channels in the paper. As per the latter, a UV lamp is used after the removal of the magnets to cure the hydrophobic portion.

Laser Treatment. Chitnis et al. [49] used a CO_2_ laser engraving machine to modulate the wettability of hydrophobic paper surfaces, obtaining porous materials for aqueous reactions. The authors tested the proposed method on commercial parchment, wax, and palette paper.

Stamping. In 2011, Curto et al. [50] proposed the use of a polydimethylsiloxane (PDMS) stamp containing hydrophobic ink as a modifying agent. The authors tested three commercial hydrophobic inks, such as black 214, noodler ink™, and black lumocolor ©, but only the latter was able to guarantee sufficient hydrophobicity. However, the resolution was low, and devices with different patterns required differently shaped stamps.

### 2.3. Detection

#### 2.3.1. Colorimetric Detection

The development of color as a result of the interaction between the analyte and one or more reagent(s) can be used in qualitative assays to evaluate the presence of a target compound. With the same approach, using a calibration curve, semi-quantitative results can also be obtained by visual evaluation.

Because of the simplicity of producing color reactions and of reading the colored products, this detection technique was soon adopted for paper-based devices. Its portability, as well as its user friendliness, with often acceptable performances in terms of sensitivity and specificity, promoted its adoption as a detection method by many researchers in this field. An additional important advantage of colorimetric paper-based devices is the possibility of carrying out simultaneous testing on a single device by means of a multichannel array. However, since, in most cases, the color reactions are specific for a class of analytes and not for a single molecule, increased selectivity can be achieved by integrating the colorimetric responses of different channels in the same array in which detection is based on different colorimetric reagents. In these cases, the application of mathematical and chemometric models, including multivariate and univariate analyses, can be a useful tool for identifying different analytes on a single-array device.

Reactions. The main strategies for producing a colored compound related to the analyte include the use of enzymes, oxidants or reducing agents, nanomaterials, and lanthanide ions. The advantages and disadvantages are strictly related to each reagent.

The use of enzyme-based reagents, in general, guarantees a highly selective reaction, and for this reason, different families of enzymes have been adopted, including oxidoreductase, nucleases, proteases, transferases, and hydrolases. The enzymes are not limited to a direct role in the catalysis of color-forming reactions in which the analyte is the substrate but are also used in enzyme-linked immunosorbent assays (ELISAs), where the specificity for the analyte depends on the immunological recognition of a molecule, while the analytical signal is generated by the enzymatic conversion of a substrate not related to the analyte. On the other hand, the use of redox reagents is grounded in the capability of the analyte to interact differently with visible light as a function of its oxidation state. The selection of the redox reaction affects the stability of the colored product [51]. Moreover, nanoparticles (NPs) are easily synthetized, functionalized, impregnated on paper, and offer a high contact surface and optical adsorption. Considering their physical–chemical properties, NPs have been integrated in biosensors for detecting toxicants in different fields, including environmental analysis, food, and biology [52]. Eventually, lanthanide ion-based reactions show an intrinsic selectivity, particularly if used in systems where selective chelation promotes the Förster resonance energy transfer (FRET) [53].

Homogeneity of the color. Although color development is theoretically invariably linked to a certain color reaction, the practical possibility of the detection of the color formation is strictly affected by the homogeneity of the produced color. In fact, poor color uniformity, the so-called “coffee ring” or “washing” effect, may hinder proper color recognition, thus resulting in poor sensitivity and reproducibility. Because of the solute-to-surface interactions along the channel and in the reaction chamber, analytes and reagents are not evenly distributed in the reaction area, resulting in color inhomogeneity, hindering the extraction of data for quantitative/semi-quantitative calculations. In order to improve color uniformity, different research groups have proposed several strategies, including paper modification, the use of nanomaterials and biopolymers, and the use of different types of paper and device designs. In particular, Garcia et al. [54] demonstrated a twofold improvement in terms of reproducibility by oxidizing and then functionalizing the paper surface with enzymes. A reduction in the coffee ring effect was also observed by adding different nanomaterials on the paper’s surface. These materials include silica nanoparticles [55], magnetic nanoparticles, multiwalled carbon nanotubes, and graphene oxide (GO) [56], as well as quantum dots (QDs), GO conjugated with Mn-doped Zn-QDs, GO mixed with Ag-NPs, and CdSe-ZnS QDs [57]. Also, the use of chitosan-based paper provided improved color uniformity [58]. Although all of the proposed strategies showed a more homogeneous color distribution, they required important efforts such as the chemical modification of a surface and the synthesis of nanomaterials. To overcome this issue, Evans et al. [59] reported that a proper selection of the paper support to optimize the flow resistance improves the uniformity of the color distribution. Martinez et al. [60] and Morbioli et al. [61] worked on the optimization reaction area, proposing a diamond shape and an origami geometry, respectively. In particular, in the former work, the authors attributed the improvement to a better dispersion of the colored product. Meanwhile, in the latter study, the authors stated that the same hydrodynamic resistance in each channel reduces the formation of privileged fluidic channels, increasing the homogeneity of the color development.

Readout and image analysis. As a result of the reaction between target compounds and reagents, the color development can be visualized by the naked eye or using an image-capturing device.

Specifically, the most straightforward approach is visualization with the naked eye, providing qualitative information based on the presence or absence of a specific color. In view of the possibility of providing semi-quantitative results without the need of dedicated instrumentation, some researchers have proposed naked-eye measurements on the basis of intensity, distance, the number of colored areas, and color development time [62]. Although these approaches are in accordance with most of the RE-ASSURED criteria and suitable for application at the point of need, to the best of our knowledge, few methods have been reported in the literature [63,64].

On the contrary, the second detection approach requires instruments for capturing an image of the device and then deconvoluting one or more color components. The main image-capturing devices can be grouped into three categories: (i) scanners, (ii) digital cameras, and (iii) smartphone cameras. Although scanners guarantee reproducible light conditions and, consequently, more reproducible results, their use requires direct contact between the device and the scanner’s surface. This requires a preliminary washing step followed by thorough drying of the device. On the other hand, image capturing with digital cameras or smartphone cameras provides high-quality results without direct contact, thus avoiding the abovementioned problems. Unfortunately, the reproducibility of the results is affected by the less reproducible lighting because of external sources. Precise color recording also depends on the reaction kinetics and of the reproducibility of the time of the image capture. In addition, smartphone cameras and the most recent digital cameras allow the operator to easily transfer images over the Internet for a remote evaluation [60]. This aspect, which is more typical of smartphone cameras, also considering their steady improvement, makes these devices the most promising for the onsite capturing of pictures.

Regardless of the image acquisition method, the area of interest is deconvoluted to extract color information using different color space systems, such as RGB (or CMYK), CIE1931, CIE L*a*b, and HSV. Specifically, the color can be considered as composed of the three colorimetric components: red ©, green (G), and blue (B) (or cyan (C), magenta (M), yellow (Y), and black (K)). According to the *Commission Internationale de l’Eclairage* (CIE), the color can be defined by parameters *x* and *y* related to the chromaticity of a color (CIE1931) [65], or, as described by CIE L*a*b, as composed by the lightness of the color (L), the red/green intensity (a), and the yellow/blue intensity (b) [66]. Alternatively, the color can be expressed as a function of the hue, saturation, and value (HSV) [67]. Although all the abovementioned color space systems have already been used for deconvoluting color in paper-based devices, because of its simplicity, the RGB system is by far the most used. However, it should be pointed out that RGB deconvolution is instrument dependent and, consequently, must be optimized for any recording device.

#### 2.3.2. Fluorimetric Detection

The fluorescence phenomenon has been used to develop paper-based devices with higher sensitivity and selectivity than colorimetric methods. The devices have been developed for chemical and biological sensing using the emission of molecules as a result of the initial electronic excitation from a light-absorption process. The main aspects that affect the fluorescence emission are the fluorophore and fluorimetric responses. In the simplest configuration, the system is conceived to obtain an emission wavelength in the visible portion of the spectrum. On this basis, the color elaboration strategies reported in the previous section are also used for fluorescence signal deconvolution.

Fluorophore. Fluorimetric detection in µPADs is based on different molecules: (i) fluorescent organic dyes; (ii) metal nanoclusters; (iii) carbon dots; (iv) quantum dots; and (v) upconversion nanoparticles [28]. Although these molecules show different advantages, their use when integrated into a paper-based device has also some cons. Fluorescent dyes, usually constituted by one or more aromatic moieties, are characterized by biocompatibility and high reactivity for surface modification but also some drawbacks in terms of self-fluorescence phenomena, small differences between the excitation and emission wavelength, and the stability of the fluorophore [28]. The unique chemical and physical properties of the metal nanoclusters have prompted several researchers to use them; in fact, these clusters are biocompatible and nontoxic and show intense emission. On the other hand, monodispersity is difficult to obtain [28]. Other materials characterized by biocompatibility are carbon nanodots, which, in addition to their easy surface modification, also have water compatibility. However, they showed a low sensitivity [28]. Finally, among the fluorophores, quantum dots (QDs) and upconversion nanoparticles (UCNPs) showed the highest Stokes and anti-Stokes shift, respectively. Also, QDs and UCNPs have high photostability, and the emission is easily tunable in QDs, while UCNPs have multicolor emissions. However, the QDs’ and UCNPs’ colloidal solutions are unstable, and QDs are toxic [28].

Fluorimetric response. The fluorescent signal produced on a paper-based device is mainly originated from a variation in intensity. The variation in the intensity has been used both as a turning-off and turning-on of the fluorescence signal; however, if applied to real samples, these approaches are more recommended for semi-quantitative measurements. In fact, the complex chemical composition of real samples may affect the fluorescence intensity, causing variations in the signal due to the matrix effects instead of the analyte concentration. On the other hand, the increase in the specificity of the interaction between the analyte and the fluorimetric probe can result in a reduced or negligible influence of the matrix components, allowing for quantitative measurements.

From a technological point of view, in comparison with colorimetric methods, the µPADs based on fluorimetric detection require the integration of other components such as optical filters and sources. In particular, the filters are used to remove the matrix components’ emission signals, which could affect the emission signal of the analyte, while the sources are used to promote the fluorescence phenomenon. In particular, for the latter, the continuous improvement of light emitting diode (LED) technology will allow for the power of excitation to continuously increase and, consequently, the intensity of emission will increase as well, allowing for improvements in the limits of detection.

#### 2.3.3. Electrochemical Detection

Undoubtedly, preliminary studies on the development of paper-based devices were mainly focused on the use of colorimetric reactions. This was justified by the inherent simplicity and affordability typical of this model of analytical devices, which, however, was plagued by several flaws and intrinsic limits hampering its practical application. To overcome these weak points, particularly in terms of sensitivity, reproducibility, and concentration range, paper-based devices using electrochemical detection have been proposed. Although this approach requires the implementation of electrodes for signal transduction in paper-based devices (µPADs), the associated advantages are sufficient to justify the increase in the complexity of fabrication, as demonstrated by the wide number of records in the scientific literature [28]. The advantages are in its easy handling, rapid response, high sensitivity and selectivity, excellent cost-effectiveness (even higher than colorimetric methods), and suitability for field analysis because of the miniaturization and portability of the main components [68,69,70]. Electrochemical paper-based devices (ePADs) measure current or voltage values, which are not influenced by the color contamination of the substrate and are independent from many variables, such as variations in the light source [71].

Since the first reported device [72], different strategies have been developed. A rapid description of fabrication methods and sensing approaches follows.

Fabrication methods. The fabrication of ePADs requires the evaluation of three aspects: (i) selection of the paper; (ii) application/modification of the electrodes; (iii) design of the device.

The types of paper platforms that can be used for designing ePADs can be roughly grouped on the basis of their adsorption capability. In particular, in chromatographic and filter papers, the intrinsic porosity of the cellulose structure affects the diffusion layer at the electrode surface. Alternatively, copy or printer papers, having a lower adsorption capability, allow for direct contact between the printed electrodes and the solution. On the other hand, the reduced porosity of the latter makes the absorption and immobilization of reagents, as well as that of electrode modifiers within the paper substrate, more complicated [73,74].

Carbon-based materials, gold, and platinum are appropriate choices for electrodes, because they are electrochemically inert over a wide range of working potentials. The embedding of electrodes in paper-based devices can be carried out by assembling conductive patterns on paper or by resorting to solid electrodes on the paper’s surface. The former uses different approaches, such as screen printing, stencil printing, inkjet printing, pen or pencil drawing [68,71,75,76,77,78,79,80], vacuum filtration, drop casting, sputtering, paper pyrolysis, and laser scribing. More recently, the use of 3D printing exploiting commercially available conductive filaments has been proposed to create flexible circuits on paper [81]. Alternatively, for the latter, carbon fibers, pencil leads, or metal wires, as well as planar electrochemical cells fabricated on a separate substrate, such as screen-printed electrodes (SPEs), can be integrated with paper platforms. Nanoparticles, such as noble metal nanoparticles (e.g., gold, silver, and platinum), metallic oxide nanoparticles (e.g., zinc, copper, or nickel oxide), and silica nanoparticles (SiNPs) have been extensively used to fabricate and/or modify the working electrodes of ePADs by directly dispersing in the ink by in situ growth, electrogeneration, or dropcasting to increase the sensitivity towards specific analytes [68,70,71,82,83]. In addition, as transduction materials, carbon-based materials have been receiving a great amount of attention in the fabrication of ePADs. These carbon-based materials include single-walled carbon nanotubes (SWCNTs), multiwalled carbon nanotubes (MWCNTs), and different types of graphene materials [84,85,86]. The use of carbon nanomaterials offers advantages in terms of high surface area, excellent electrical conductivity, and high surface reactivity, thus facilitating not only the transfer rate of electrons but also providing a large surface for the immobilization of biomolecules and recognition elements. In fact, molecularly imprinted polymers (MIPs), enzymes, aptamers, nanozymes, and antibodies have been added to the sensor, obtaining an increment in selectivity while maintaining other advantages, including easy production at a large scale, low cost, and high stability [87,88]. Improvements in the detection performance have also been observed by using metal–organic frameworks (MOFs) [89] and very highly conductive polymers such as polyaniline (PANI) [84].

As per the device design, planar (2D design) and vertical (3D design) flows can be used. Bidimensional devices based on fluids with different designs and geometries or more complex 3D architectures based on multidimensional structures with multilayer connections between paper layers and assembled by folding, bending, and twisting have been coupled to EC detection. To this aim, origami devices [90], assembled by paper folding, offer the advantage of the vertical diffusion of the liquids through the different paper layers, which can be exploited for purification, preconcentration, or derivatization of the sample before reaching the surface containing the electrochemical transducer. In addition, hollow channels, where the cellulose matrix is physically removed from the channel, introducing convection to the microchannel [91,92] and reducing absorption phenomena, can be used. In fact, the cellulose matrix, acting as a barrier to diffusion, limits convection and partially blocks part of the electrode surface. In some cases, ePADs can be employed in the sample preparation or preconcentration to remove interferences and increase the selectivity also in combination with other detection approaches.

Sensing techniques. In general, electrochemical techniques can be classified as follows: (i) amperometric/voltametric, (ii) potentiometric/conductometric, and (iii) impedimetric/capacitive [93,94,95,96].

Among these approaches, amperometric techniques, where the current is measured at a given potential (amperometry or chronoamperometry) or during a potential scan (voltammetry) offer merits that include sensitivity, detection limits up to 10^−12^ M, low costs, ease of use, and, sometimes, selectivity, in comparison with alternative approaches such as potentiometric electrochemical sensors. In general, a potential is applied to the working electrode and, consequently, electrochemical reactions involving the analyte occur at the electrode interface, where the resulting current is monitored and can be correlated with the analyte concentration. Amperometric approaches have been coupled to injection analysis either under flow (FIA) or in static systems (BIA, batch injection analysis), as well as coupled to paper-based separation systems [75,97]. These devices were assembled using cellulose supports exploiting paper chromatography with pump-free fluid transport across the cellulose matrix via capillary action. Unmodified and modified paper using anion-exchange modifiers or large-pore silica gel have been used [98].

In voltammetry, a linear scan is performed (cyclic voltammetry or linear sweep voltammetry) or, as in differential pulse voltammetry (DPV) and square wave voltammetry (SWV), pulses are superimposed. The current is sampled before and after the pulse in DPV or before the end of forward/backward pulses in SWV, thus decreasing the capacitive current and, in turn, increasing the sensitivity.

Different configurations based on two (working and counter) or three electrodes, including integrated and stable reference electrodes, have been proposed [82,83,85,99,100]. In addition, systems including two or more independent working electrodes, arrays, or IDEs assembled in the same or on the two opposite faces of the paper have been proposed for multiplexed determinations [101,102,103,104,105], selective detection of analytes displaying reversible behavior, or signal amplification exploiting redox cycling. At the same time, the use of microelectrodes has been integrated with paper-based analytical devices in view of improving sensitivity [106].

#### 2.3.4. Spectroscopic Detection with a Focus on Surface-Enhanced Raman Scattering (SERS) Spectroscopy

Surface-enhanced Raman scattering (SERS) spectroscopy aids in the identification of analytes, potentially down to the single-molecule level, due to the extremely high selectivity of Raman scattering. SERS spectroscopy is based on the collection of light scattered inelastically from a sample when illuminated with a laser in the presence of noble metal nanostructures (NMNs), generally called “SERS substrates”, commonly made of gold or silver [107]. SERS substrates are used as signal amplifiers to enhance the weak Raman signal, providing sensitivity comparable to fluorescence spectroscopy but also delivering highly specific information about the analyte. The molecule’s ability to adsorb in the interstices next to the NMNs, or “hot spots”, causes the strength of their SERS signal to increase because of the strong electromagnetic field associated with the surface plasmon resonance (SPR) of NMNs. This allows for the identification of specific molecules with unique recognizable spectral bands, even in complex mixtures [108].

The use of noble metal nanostructures (NMNs) in paper-based SERS substrates to enhance the Raman signal has various benefits in terms of price, stability, simplicity, and fabrication methods [109]. Paper’s porosity offers a huge surface area for assembling nanostructures in high densities. With its built-in capillary action, cellulose filter paper provides several chances for chemically or physically regulating the deposition of NMNs and the capture of analyte molecules throughout a range of pore widths or special pore dimensions [110,111,112]. It is worth mentioning that the Raman signal of cellulose is negligible, which would reduce the interference of the background when detecting various types of targets [113]. In addition, paper-based devices offer the advantage of portability over conventional colloidal SERS substrates and can be utilized directly with the handheld Raman analyzers.

In the most typical presynthesized substrate approach, NMNs, like nanoparticles (NPs), nanostars (NSs), or nanorods (NRs) of silver (Ag), gold (Au), or copper (Cu), are deposited on paper by simply dipping, spraying, gravity-filtration, immersion, or soaking [114]. Moreover, by employing colloidal nanoparticles, such as inks, pen-writing or inkjet printing techniques offer an additional universal method for depositing nanostructures of noble metals onto paper and developing flexible and tunable sensing devices [115,116,117].

While untreated paper can be used for many SERS applications because of its low cost, ease of fabrication, and scalability, hydrophobic treatment has attracted a lot of attention in recent years as a means of improving paper for use in SERS platforms [76,118,119]. The main reason for this is to prevent the aqueous analytes from spreading too far across the paper substrate, therefore reducing the contact concentration with the hot spots. This can improve the SERS response in many cases, albeit a systematic evaluation of the impact of the interaction among paper, substrate, and analytes is still missing.

Direct and indirect detection. In general, SERS detection works effectively with analytes that have either a directly binding moiety (such as a thiol functional group) or a high affinity for NMNs’ surfaces; however, the analysis and detection of nonbinding compounds can be significantly more difficult, especially if the matrix in which the specific analyte of interest is found is chemically complex (e.g., biofluids). On the other hand, to avoid problems related to interference from the competitive adsorption of other molecules present in the biological media, “indirect detection” is used. Variations in the SERS signal of other molecules (Raman reporters or probes) are used to infer the existence or quantity of the analyte of interest. For example, “SERS nanotags” can be created by binding Raman reporters and recognition elements (e.g., antibodies) to NMNs [120] and are often used as labels in paper-based lateral flow strips. The detection sensitivity of these devices is thus determined by the intensity of the SERS nanotags [114].

Quantitative analysis. Absolute quantification with SERS, like with any other measurement method, requires that the system is properly calibrated. For practical applications, the intensity of the SERS signal should be proportional to the number of molecules adsorbed to the substrate surface. However, the intensity of the SERS signal is determined by how many molecules are actually in the hot spots, and a slight variation in the geometric structure, such as interparticle distance or molecule diffusion, may result in a signal change by several orders of magnitude. Several additional factors can also play a role, such as the orientation of adsorbed molecules and how the laser beam changes during the experiment. It is worth noting that the uniformity of SERS signal on µPADs is significantly impacted by the size of the laser spot. Better results with increased precision can be obtained by employing a larger laser spot because of an averaging effect. This intrinsic variability caused by the substrates may be reduced with the use of internal standards, such as isotopologues or by the standard addition method [121]. Core–shell NMNs, embedding the internal standard molecules, have also been proposed [122]. However, the complicated preparation of core–shell substrates has limited extensive applications, making this kind of µPADs impractical to manufacture.

## 3. Applications

The determination of drugs and toxicants is the widest area of forensic science where the use of paper devices has been proposed. Although most of these molecules are also relevant in different fields, such as environmental and food analysis, the present report is mainly focused on the toxicological analysis of biological fluids, offering a critical evaluation of the performances of paper-based devices in terms of sensitivity, selectivity, and reproducibility. Also, the stability of the devices was evaluated, since it is a fundamental requirement for their deliverability to the end user, considering the difficult context for which they were intended.

The following section is divided into three parts:

- Toxic compounds (arsenic, cyanide, ethanol, and nitrite);
- Drugs and drugs of abuse (benzodiazepines, cathinones, cocaine, fentanyl, ketamine, MDMA, morphine, synthetic cannabinoids, and tetrahydrocannabinol);
- Psychoactive substances used for drug-facilitated crimes (flunitrazepam, GHB, ketamine, metamizole, midazolam, and scopolamine).

### 3.1. Toxic Compounds

Arsenic. Although its use for murder or suicide has nowadays drastically dropped in comparison to past centuries, arsenic compounds are still used in illicit actions for their harmful though inapparent action [123,124]. The toxicity of arsenic can be attributed to various mechanisms. In particular, it inhibits the first step of gluconeogenesis by strongly binding the cofactor of pyruvate dehydrogenase. Also, arsenic competes with phosphate for the reaction from adenosine diphosphate to ATP. It binds to proteins, distorting their three-dimensional configurations, and it has also been suggested that the toxicity of arsenic is caused by its ability to bind to enzymes involved in the heme biosynthetic process [3].

Very recently [125], it was demonstrated that arsenic promotes a structural change in silver nanoprisms, resulting in a color change. The proposed paper-based device strategy was used for detecting arsenic in urine, showing a limit of quantification comparable to or even better than the currently used devices (LOQ = 0.5 µg/L). The authors partially validated the device by testing its stability, specificity, and reproducibility. Although the device can be used to detect the analyte after 8 days of the device preparation, the other two parameters tested represent two main weak points of the present device. In fact, most of the tested ions interfere with the analyte detection, and the aggregation process suffers, according to the authors, from the random fibrous structure of the paper.

Cyanide. Cyanide in the blood stream passes through biological membranes and strongly binds to the iron in the mitochondrial cytochrome a-a3 complex. This binding results in the inhibition of cytochrome oxidase, causing hypoxia, respiratory disorders, and eventually death. The determination of cyanide is commonly based on the use of microdiffusion devices [126] and/or chromatography [127,128]. Unfortunately, these methods require time-consuming laboratory procedures and/or benchtop instrumentation.

**Figure 1:**
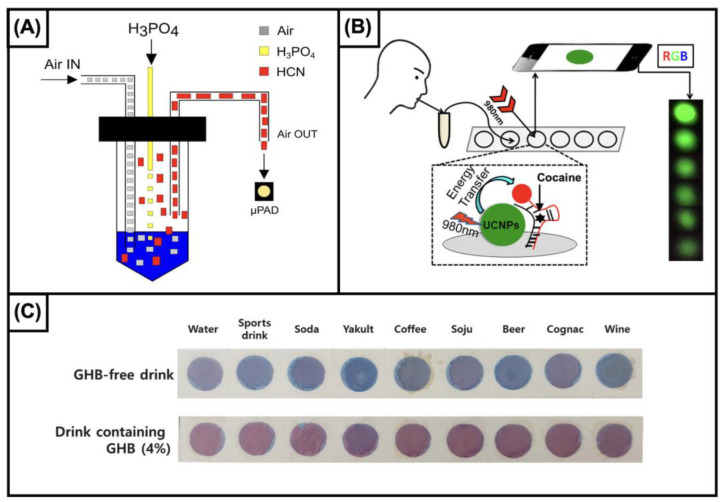
(**A**) Schematic representation of cyanide determination. A homemade device has been designed to detect HCN at the gas phase. The liquid sample is gently insuffled with air, and added with phosphoric acid (30%). The detection was carried out using palladium-dimethylglioximate. “Reprinted from Sensors and Actuators B: Chemical, 268, da Silveira Petruci, J.F. et al., Colorimetric paper-based device for gaseous hydrogen cyanide quantification based on absorbance measurements, 392-7, Copyright (2023), with permission from Elsevier”. (**B**) Graphical illustration of the procedure for detecting cocaine in saliva samples. The device uses up-conversion nanoparticles (UCNPs) functionalized with anticocaine aptamers. In the presence of cocaine, the UCNPs luminescence is quenched. The luminescence change can be observed by naked eye for qualification or recorded by a smartphone camera. “Reprinted (adapted) with permission from Analytical Chemistry, 88, He, M. et al., Portable Up conversion Nanoparticles-Based Paper Device for Field Testing of Drug Abuse, 1530-4, Copyright (2023), American Chemical Society”. (**C**) Image of the interaction between the colorimetric reagent developed for detecting GHB with common drinks, and with the same drinks spiked with the analyte. The proposed device is based on the interaction between GHB and pentacosadiynoic acid-gabazine reagent which turns from blue to red. “Reprinted from Sensors and Actuators B: Chemical, 347, Son, S.U. et al., Colorimetric paper sensor for visual detection of date-rape drug γ-hydroxybutyric acid (GHB), 130598, Copyright (2023), with permission from Elsevier”.

In 2018, Petruci et al. [129] proposed the integration of a gas diffusive strategy with a paper-based device with the aim of optimizing selectivity by determining the gaseous form of the analyte and affordability by miniaturizing the device and the need for reagents (Figure 1A). The authors tested a homemade tool integrating not only the gas diffusion step and the paper-based device but also the detection system. In particular, the color resulting from the interaction between the analyte and palladium dimethylglyoximate was processed with an LED-based detector embedded in the same system. A limit of detection of 10 µg/L was reported. Unfortunately, notwithstanding the fact that the authors had proposed the use of this device for detecting cyanide in liquid samples, including blood, no results on biological matrices were reported. More recently, Sheini et al. [130] proposed a multilayer (*n* = 5) paper-based device for detecting cyanide from blood samples based on sequential reactions for cyanide separation from iron and then detection. The development of the device included the optimization of the sample volume, detection conditions, reaction time, and reagent concentration. The authors did report the selectivity and the reproducibility of the device and, more interestingly, used it to test different blood samples, which included samples from smokers and nonsmokers from the general population, firemen (five each), and people involved in fire cases who did or did not survive (five each). The results were compared with those obtained with GC-MS, showing good concordance in terms of accuracy. The study, although promising, of course, requires a more representative number of analyses of real samples and a thorough validation.

Another interesting approach to the determination of cyanide is the determination of thiocyanate in urine. In fact, approximately 80% of cyanide is metabolized to thiocyanate and excreted into urine [25]. To the best of our knowledge, the literature on the use of paper-based technology for detecting thiocyanate in urine is limited. In 2021, Wirojsaengthong et al. [131] proposed the detection of thiocyanate using a cobalt porphyrin derivative. The selectivity of the device was tested against 11 ions (fluoride, chloride, bromide, iodide, nitrite, nitrate, sulphate, dihydrogenphosphate, perchlorate, arsenite, and arsenate), and only nitrite gave a low-intensity interfering signal. The device was tested for the analysis of spiked urine samples, showing an accuracy higher than 85% and limits of detection with the naked eye and a smartphone camera of 2.9 mg/L and 0.073 mg/L, respectively [131]. The urine of workers chronically exposed to cyanide showed concentrations ranging from 1.5 to 12.9 mg/L for nonsmokers (*n* = 15) and 1.5 to 16.5 mg/L for smokers (*n* = 8) [25].

Ethanol. In Western countries, ethanol is the most used and abused psychoactive compound. The range of acute toxicity of ethanol spans from blood concentrations of 0.5 mg/L up to above 5 mg/L.

Although the analysis of ethanol is one of the most widespread tests, using paper-based device technology to detect ethanol in biological fluids has been proposed only recently [132]. Thepchuay et al. [132] developed a colorimetric test for detecting ethanol using an enzymatic reaction. The method was preliminarily tested using mice and sheep blood, showing suitable sensitivity and specificity for detecting the analyte in complex biofluids. The selectivity was guaranteed by the integration of a gas diffusion strategy and by the use of a specific reaction based on two enzymes. In addition, the small sample volume required (8 µL), the small size of the device, and its affordability, after proper validation on human blood samples, could make the device suitable for onsite measurements [132]. Paper-based technology for detecting ethanol has also been integrated with cerium (Ce) nanoparticles in a homemade breath analyzer device. The formation of a yellow color [Ce(III) → Ce(IV)] was the result of the formation of hydrogen peroxide through the enzymatic reaction alcohol oxidase. Although the approach approximately showed the same dynamic concentration range as the previous method (blood concentrations ranging from 0.2 to 1.2 g/L), the analytical sensitivity was highly improved. This improvement was needed because of the much lower concentration of ethanol present in the breath, approximately 1/2100–2300 of the blood ethanol concentration [3].

Nitrite. This inorganic anion promotes the oxidation of iron (II) contained in hemoglobin with the formation of methemoglobin, which has a limited capacity of transporting oxygen. The resulting hypoxia causes severe effects as a function of the percentage of methemoglobin, such as skin discoloration (<20%), fatigue and syncope (20–50%), coma (50–70%), and death (>70%) [25].

Among the compounds included in this report, nitrite is the most investigated molecule in the development of rapid approaches using paper-based technology (Table 2). This is mainly due to several diseases in which nitrite is involved, including sepsis, infectious gastroenteritis, meningitis, and periodontitis.

However, it must be reported that the determination of nitrite has also recently gained importance in forensic toxicology. In fact, in several cases of fatal or nonfatal intoxication, nitrite has been used, either accidentally or deliberately, in suicidal or homicidal contexts [133]. Most of the rapid methods for the direct detection of nitrite using paper-based devices have so far been performed using the Griess colorimetric reaction, or a modified version of the reaction [36,134,135,136,137,138] in which citric acid was replaced by phosphoric acid or hydrochloric acid [139,140,141,142]. Because of the importance of a high sensitivity, the approach proposed by Zhang et al. looks particularly interesting because the authors, integrating an electrokinetic stacking mechanism, increased the analytical sensitivity of the device up to 160-fold, which was able to detect nitrite down to 0.9 µmol/L [134].

Very recently, Hou et al. proposed a new method based on metal–organic frameworks (MOFs) for the chemiluminescence (CL) detection of nitrite in blood. The MOF structure catalyzes the conversion from nitrite to oxygen peroxide for CL detection. The detection limit was in the order of subnanomolar concentrations, i.e., 0.5 nM [143]. Although this proof-of-concept work showed interesting characteristics, such as nanomolar sensitivity and suitability for analysis of blood, the complexity of synthesizing the MOF system and the lack of a full validation dramatically limit its practical value.

### 3.2. Drugs (D) and Drugs of Abuse (DoA)

Benzodiazepines (D). Benzodiazepines act as depressants of the CNS and are classified on the basis of the duration of their effect into short-acting, intermediate-acting, and long-acting drugs. In this family, paper-based devices have been developed for alprazolam and diazepam.

Alprazolam. Two different devices have been proposed for detecting alprazolam in different matrices, such as vitreous humor, blood, and urine. The determination of alprazolam in blood and vitreous humor has been carried out using a colorimetric detection based on the analyte-promoted aggregation of silver nanoparticles. Although authors have reported sensitivity in the order of submicrograms per milliliter (0.8 µg/mL), the procedure required a liquid–liquid extraction, limiting its use at the point of need [144]. This limit has been overcome by the device proposed by Narang et al., who reported a determination range of 1–300 ng/mL in spiked urine with an LOD of 0.025 ng/mL. The determination was performed using a modified electrode made of methylene-blue-doped silver core–shell palladium (Ag@Pd). By means of methylene blue as a redox transition substance, alprazolam was reduced to the corresponding dihydro derivative, and this led to a selective DPV signal related to the analyte [82].

Diazepam. The determination of diazepam was achieved by means of silica-coated gold nanorods (Si@GNRs) dropcasted on an electrochemical microfluidic paper-based device. The electrochemical sensing of diazepam was carried out on a two-electrode system using cyclic voltammetry and electrochemical impedance spectroscopy and was able to sense diazepam in spiked urine samples in a range spanning from 1 µg/L to 1 g/L, with an LOD of 0.42 µg/L.

Unfortunately, these studies lack proper validation. In particular, limited or no information on the selectivity are available, and none of the reports included data on precision and accuracy [82,83,144].

Cathinones (DoA). Cathinones are designer stimulants with effects on the central and peripheral nervous systems as a result of blocking dopamine and serotonin reuptake.

Mephedrone. Recently, McNeill et al. developed an origami paper-based device for detecting mephedrone and its main metabolite in urine [90]. Although colorimetric sensing cannot be used for quantitative measurements, it can effectively be used for a qualitative determination, with sensitivities in the order of few nanograms per milliliter (i.e., 4.34 ng/mL). Another point to highlight is the capability to also detect similar synthetic cathinones and amphetamines. The cross-reactivity of the device was tested for adulterants (amphetamine, cocaine, and ketamine), cutting agents (benzocaine, caffeine, lidocaine, paracetamol, procaine, and taurine), and other potential interferents (corn flour and flour), proving no statistically significant variations in the signal [90].

Cocaine (DoA). Cocaine is a natural psychoactive alkaloid extracted from *Erythroxylum coca* leaves. It acts on the inhibition of dopamine and noradrenaline reuptake, provoking a well-known stimulating and euphoric effect.

The determination of cocaine in biofluids using paper-based devices has been proposed using highly selective recognition elements, i.e., aptamers. Yang’s research group proposed two approaches using aptamers as stabilizing agents of a hydrogel structure. The authors highlighted the high selectivity of the devices, showing a limited interaction with the main metabolites of cocaine. Although the two strategies proposed by Yang et al. were based on the same principle, for the earlier approach [145], the release of a food colorant as a result of the analyte contained in the sample allows for the detection of cocaine up to 15.15 ng/mL, whereas the cascade reactions strategy [146] reported in the second paper resulted in the improved sensitivity of one order of magnitude (3 ng/mL). Also, the latter approach was successfully tested for semi-quantitative measurements, while the former was only tested for qualitative analyses. However, the integration of aptamers and hydrogel allowed for the detection of cocaine in biofluids, such as urine, even in the order of a few nanograms per milliliter. For toxicological purposes, other matrices, such as blood or saliva, which better reflect the action of cocaine, are of higher interest. For this purpose, the use of devices based on aptamers was proposed, also integrating upconversion nanoparticles (UCNPs) and gold nanoparticles (AuNPs) [147]. In particular, the authors developed a device for detecting cocaine in saliva based on two single-strand anticocaine aptamers (ACA-1 and ACA-2), obtaining a sensitivity as low as 3 ng/mL. Also, the same strategy provided satisfactory results for analyzing cocaine in rat plasma. In this device, the integration of recognition elements allowed for the selective detection of cocaine but sacrificed the detection of its metabolites (Figure 1B).

The detection of cocaine has also been largely investigated using paper-based SERS devices, but most of these approaches were tested in pure solutions [76,116,148,149]. Of note, Burr et al. integrated a portable Raman spectrometer and a portable ion trap MS system [149] showing limits of detection of 0.6 and 13 ng, respectively (Table 3). Interestingly, the dual SERS-PSI-MS platform was validated in a blinded error rate study (*n* = 500) and resulted in a 99.8% accuracy, with no false positives observed. To the best of our knowledge, the determination of cocaine in complex biofluids using a paper support for SERS detection was proposed in only a single report. The authors proposed the use of a plasma-printed paper substrate to detect the analyte in spiked saliva, investigating the range from 1 (limit of detection) to 5000 ng/mL. The sensitivity was suitable for detecting cocaine in real samples; however, a liquid–liquid extraction was required to detect cocaine close to the detection limit.

Fentanyl (D). Fentanyl is a synthetic opioid used in applications ranging from surgical anesthesia to the management of acute and chronic pain [3]. Its well-known side effects include severe respiratory depression, muscle rigidity, coma, and hypotension [25].

To the best of our knowledge, in 2021, the determination of fentanyl in biofluids using a paper-based device coupled to an SERS device was proposed for the first time. Han et al. [150] tested the device on artificial urine and rat serum. The method was validated yielding recovery rates of 92.0–101.1% and 90.9–103.2%, respectively. The proposed device was capable of detecting fentanyl in artificial urine and rat blood down to 0.59 μg/mL and 2.78 μg/mL, respectively. Although interestingly, urine and blood concentrations usually found in fatal cases are in the order of 3–28 ng/mL in blood and 5–93 ng/mL in urine [25]. This scarce sensitivity clearly represents a limit in real cases of intoxication.

Although other published reports were proposed for analyzing surfaces [151] and pure solutions [116,149,152], these methods also seem to be important for forensic toxicology, since they describe tools to protect the laboratory personnel from fentanyl toxicity by inadvertent contact [25]. The strategy proposed by Haddad et al. was tested on absorbent and nonabsorbent surfaces, showing a submicrogram limit of detection [151]. Tay et al. proposed an improvement in the sensitivity to 10 ng/mL via iodide functionalization [116,152]. On the other hand, Burr et al. improved the detection limit to 1 ng per microliter of solution using an instrument integrating SERS and MS detection [149].

Ketamine (D). In pediatric medicine, ketamine is used as an anesthetic for short surgical procedures. However, it can cause dissociation of consciousness, perception, and movement in a short period of time. It is regarded as a dissociative anesthetic because of its ability to antagonize the excitatory system. Ketamine is also abused, particularly by health system personnel. Finally, it is often used in so-called drug-facilitated crimes since aggressors can easily conceal the ketamine in powder or liquid form to discreetly administer it to the victim.

The detection of ketamine in oral fluid using a paper-based device was reported using a competitive enzyme-linked immunosorbent assay (cP-ELISA). The method was tested within the range 0.001–1000 ng/mL, showing linearity from 1 to 1000 ng/mL and a limit of detection of 0.03 ng/mL. The approach was applied to the analysis of 90 real samples, and the results were compared with a validated GC-MS method. The diagnostic sensitivity and specificity were 90% and 92%, respectively [153].

MDMA (DoA). Methylenedioxymethamphetamine is a designer drug related to phenylethylamine, which is widely used as a recreational drug [25]. Moreover, it is also typically associated with date-rape crimes [154]. MDMA has a stimulant effect on the CNS, causing sympathomimetic effects, including peripheral vasoconstriction, bronchodilation, cardiorespiratory stimulation, pupillary dilation, and appetite suppression, but its most “liked” effect is “entactogenic”, i.e., facilitating social interactions [3].

So far, the only determination of MDMA using a paper-based device has been reported by Narang et al., who proposed a two-electrode paper device integrating ZnO nanorods obtained using a hydrothermal synthesis [100]. The device was designed to detect MDMA in urine and sweat down to 19 ng/mL, and the response was linear up to 190 µg/mL, showing accuracy and recovery ≥ 90%.

Morphine (D). Morphine is an opioid classified as a narcotic analgesic which can cause respiratory depression, decreased reflexes, bradycardia, hypotension, decreased intestinal motility, hypothermia, miosis, pulmonary oedema, and coma [3].

Morphine is one of the first drugs for which a paper-based device was proposed. In 2014, Teerinen et al. [155] developed a paper-based lateral flow assay to detect morphine in spiked oral fluid. The assay was based on a direct sandwich approach, in which the immunocomplex composed of morphine and the anti-morphine Fab fragment was detected using another Fab fragment. The method showed a sensitivity of 20 ng/mL (LOD). The authors tested its performance by comparing the assay results with those obtained using a common nitrocellulose-based test and by estimating the imprecision of the method. However, the reference approach is based on the same principle, and the imprecision of the method varied from 10 to 28%, limiting its use in forensic toxicology contexts [156].

Synthetic cannabinoids (DoA). Synthetic cannabinoids include a family of over 200 compounds that interact with cannabinoid receptors, causing adverse effects, including tachycardia, hypertension, and altered time perception [25].

Moulahoum et al. integrated a rhodamine B-loaded polymersome in a paper-based device for detecting JWH-073 (one of the first compounds released on the market) in saliva [157]. The authors optimized the device to carry out both colorimetric and fluorimetric detection, obtaining LODs in the order of at subnanogram per millimeter levels (colorimetric sandwich, 0.53 ng/mL; colorimetric competitive, 0.31 ng/mL; fluorescence, 0.16 ng/mL). The device showed repeatability and reproducibility equal to or better than 5.6%, either in synthetic or real saliva. Also, the approach was validated in terms of selectivity vs. other drugs, such as cocaine, amphetamine, methamphetamine, and tetrahydrocannabinol, and the anti-K2 antibody-based polymersome showed a high interaction only for the analyte [157].

Tetrahydrocannabinol (DoA). Tetrahydrocannabinol (THC), better named ∆^9^-THC, ∆^1^-THC, is the main active component of cannabis sativa. As universally known, its intake, in most cases by smoking, causes sedation, euphoria, and hallucinations [25].

So far, the only available method for detecting THC with a paper-based device is based on electrochemical analysis. Pholsiri et al. [86] reported the use of a screen-printed graphene electrode modified with copper phthalocyanine (CuPc), which was applied to THC analysis in saliva. The device was able to detect the cannabinoid compound within the range of 10–1500 ng/mL with an LOD of 1 ng/mL. The selectivity was tested on samples containing different ions and small molecules, namely, chloride, nitrate, acetate, carbonate, nitrite iodide potassium, dihydrogenphosfate, sodium, magnesium, sulfate and sulfite, citric acid, glucose, cysteine, and ascorbic acid [86].

### 3.3. Psychoactive Substances Used for Drug-Facilitated Crimes (DFCs)

In addition to strategies for detecting psychoactive drugs in biological fluids, in the specific context of DFCs, efforts have been made to realize the rapid toxicological analysis of beverages suspected to contain DFC compounds. The list of sedative drugs suitable for this purpose include some benzodiazepines (e.g., flunitrazepam, midazolam, diazepam, temazepam, clonazepam, and oxazepam), as well as gamma-hydroxybutyrate (GHB), ketamine, MDMA (see above), and scopolamine.

So far, the use of paper-based devices has been reported for the analysis of flunitrazepam, GHB, ketamine, metamizole, midazolam, scopolamine, and xylazine.

Flunitrazepam. Tantawy et al. [96] fabricated a paper-based device using silver and carbon ink with the application of poly(3,4-ethylenedioxythiophene) (PEDT) nanoparticle dispersion on the electrode surface. The optimization of the device allowed the detection of flunitrazepam down to 0.17 mg/L. The device was also validated in terms of trueness, imprecision, and robustness, showing values of 98%, 1.77%, and 0.57%, respectively. Its practical application for detecting flunitrazepam was tested in Coca-Cola^®^, Fanta^®^, Sprite^®^, Red Bull^®^, Barbican^®^, and Nestle^®^ Purelife^®^ water, showing no interferences.

GHB. Undoubtedly, among the compounds included in this paragraph, GHB represents the most “famous” molecule illicitly used for committing drug-facilitated crimes. Although the ingestion of GHB causes euphoria, increases sexual desire, has a CNS depressant effect, and enhances growth hormone release, it has been mainly used for illegal purposes because of its rapid onset and hypnotic and short-term amnestic properties [3]. Worthy of note is a recent approach proposed by Son et al. [158]. The authors developed a paper sensor based on the use of a pentacosadiynoic acid–gabazine reagent. They demonstrated the capability of the sensor to effectively detect the analyte in different beverages, such as water, sports drinks, soda, yakult, coffee, soju, beer, cognac, and wine. Also, to facilitate the use of the device, the authors implemented a smartphone-based approach to easily identify adulterated beverages (Figure 1C).

Although the proposed strategy could offer a suitable device for detecting GHB illegally added to beverages, the complexity of the synthesis of the reagent limits its design to highly specialized laboratories. The simplification of the synthetic procedure or the commercialization of the reagent could increase the use of the approach.

Ketamine. Different strategies for detecting ketamine added to beverages are present in the literature. In 2020, Yehia et al. [159] proposed a trimodal system to detect and quantify ketamine in beverages. The three-way sensing strategy included colorimetry, fluorimetry, and potentiometry, reaching sensitivities of 10, 0.048, and 0.0008 g/L, respectively. The determination of ketamine using paper-based devices was also proposed by Narang et al. using colorimetry [63] and electrochemistry [85]. The colorimetric paper-based device for detecting ketamine in beverages showed a detection limit of 2.4 g/L, being tested on cola, rum, and whiskey [63]. The latter device used a two-electrode system made of nanocrystals of graphene-oxide and zeolites integrated on a working electrode [85]. Ketamine determination was carried out on soft and alcoholic drinks using cyclic voltammetry (CV) and electrochemical impedance spectroscopy (EIS). The nanocrystal-modified EμPAD showed a wide linear range of 1–3000 µg/L and a detection limit of 0.2 µg/L [85].

Metamizole and Midazolam. In 2018, Dias et al. [77] optimized a pencil-drawn electrochemical paper-based device for detecting midazolam and metamizole in whiskey samples. Using square wave voltammetry, the device could detect midazolam and metamizole concentrations as low as 5 mg/L and 20 mg/L, respectively. In 2021, a similar analytical device was developed based on a sandpaper substrate, which detected metamizole maleate concentrations as low as 3 mg/L [78].

Scopolamine. This tropane alkaloid, already known for its incapacitating agent and inducing the submissive behavior of the victim, has been recently found in a drug-facilitated crime case [160]. Dias et al. [161] proposed a multiplex device for detecting scopolamine, atropine, cocaine, morphine, ephedrine, caffeine, dipyrone, and alprazolam for the presumptive identification of drug-adulterated beverages. The authors used two different independent unsupervised techniques, namely, principal component analysis (PCA) and hierarchical cluster analysis (HCA), to distinguish the eight alkaloids, tentatively showing the device’s capability to discriminate similar compounds. However, tests on real samples, such as spiked gin, vodka, sugarcane spirit, and white wine, have only been carried out on scopolamine.

Xylazine. Xylazine is a veterinary sedative, which, for most intoxication cases, is related to accidental consumption or abuse aims, and causes effects including bradycardia, respiratory depression, and, in some cases, death [25].

Saisahas et al. [84] proposed an electrochemical device for xylazine detection integrated with a smartphone. A three-electrode system was deposited on paper using graphene and Ag/AgCl inks. Coral-like polyaniline was used to dope the WE, providing an electron transfer medium with a large surface area. The sensor was tested directly on xylazine and led to two linear responses: from 0.2 to 5 mg/L and from 5 to 100 mg/L, with an LOD of 0.06 mg/L.

**Table 2:** Paper-based devices proposed for clinical and forensic toxicology issues. The explanation of the principle on which is based each device is included in the Supplementary Materials file.

Category	Analyte	Matrix	Detection (Sensing Molecule)	Equipment (Detection System)	Limit of Detection	Linearity	Comparison with a Different Method	Ref.
Detection of Toxic Compounds in Biofluids	Arsenic	Urine	Colorimetric (AgNPr)	Qualitative determination with the naked eye	0.5 µg/L (6.7 nmol/L)	0.5–1000 µg/L (6.7–13,000 nmol/L)	Spectrophotometric method (UV-Vis)	[125]
	Cyanide	Liquid samples	Colorimetry (Pd dimethylglioximate)	Homemade device integrating air flow module, addition of acid and LED-based detector (LED detector and laptop for signal elaboration)	10 µg/L (0.4 µmol/L)	n/a	GC-MS	[129]
	Cyanide	Blood (plasma)	Colorimetry [Pt complex ([Pt(p-MeC6H4)2(phen)])]	Centrifuge (smartphone and laptop for signal elaboration)	10 µg/L (0.4 µmol/L)	26–2600 µg/L (1.0–100 µmol/L)	GC-MS	[130]
	Thiocyanate (metabolite of cyanide)	Urine	Colorimetric (cobalt porphyrin derivative)	(smartphone and laptop for signal elaboration)	73 µg/L (1.26 µmol/L) (smartphone); 2.9 mg/L (50 µmol/L) (naked eye)	1–100 µmol/L	Ion chromatography	[131]
	Ethanol	Blood (mice blood, sheep blood)	Colorimetric (ABTS)	(camera and laptop for signal elaboration)	0.12 g/L (2.6 mmol/L)	0.12–1.2 g/L (2.6–26 mmol/L)	HS-GC-MS	[132]
		Breath	Colorimetric (cerium nanoparticles)	(smartphone and laptop for signal elaboration)	0.01 g/L (0.2 mmol/L)	0.2–1.2 g/L (4–26 mmol/L)	Electronic breathalyzer	[162]
	Nitrite	Saliva	Colorimetric (Griess reagent)	Centrifuge, circuit for generating voltage (smartphone)	75 µg/L (1.6 μmol/L)	75–1000 µg/L (1.6–21 μmol/L)	n/a	[134]
		Artificial urine	Colorimetric (Griess reagent)	(smartphone and laptop for signal elaboration)	2.3 mg/L (50 µmol/L)	n/a	n/a	[135]
		Saliva	Colorimetric (modified Griess reagent—phosphoric acid)	Nitrogen to store the device (scanner and laptop for signal elaboration)	0.46 mg/L (10 µmol/L)	0.46–46 mg/L (10–1000 µmol/L)	Spectrophotometric method (UV-Vis)	[139]
		Saliva	Colorimetric (modified Griess reagent—phosphoric acid)	(scanner and laptop for signal elaboration)	7.8 µg/L (0.17 µmol/L) (nitrite); 16.7 mg/L (0.27 mmol/L) (nitrate)	0.23–11.5 mg/L (5–250 µmol/L) (nitrite); 9.2–55.2 mg/L (0.2–1.2 mmol/L) (nitrate)	n/a	[140]
		Saliva	Colorimetric (Griess reagent—citric acid)	(smartphone, photo box equipped with 84LEDs and laptop for signal elaboration)	0.46 mg/L (9.6 µmol/L); 3.4 mg/L (74 µmol/L)	0.92 mg/L (0.02–5 mmol/L)	n/a	[136]
		Saliva	Colorimetric (modified Griess reagent—hydrochloric acid)	(scanner and laptop for signal elaboration)	0.26 mg/L (5.6 µmol/L)	0.26–1.15 mg/L (5.6–25 mmol/L)	Spectrophotometric method	[141]
		Saliva (artificial)	Colorimetric (modified Griess reagent—hydrochloric acid)	(smartphone and laptop for signal elaboration)	0.26 mg/L (5.7 µmol/L)	0.26–46 mg/L (5.7–1000 µmol/L)	HPLC-UV	[142]
		Urine/serum	Colorimetric (Griess reagent—citric acid)	(smartphone and laptop for signal elaboration)	0.2 mg/L (4.3 µmol/L) (serum); 0.1 mg/L (2.3 µmol/L) (urine)	0.23–27.6 mg/L (5–600 µmol/L) (serum); 0.23–4.6 mg/L (5–100 µmol/L) (urine)	n/a	[137]
		Saliva	Colorimetric (Griess reagent—citric acid)	(smartphone and laptop for signal elaboration)	1.15 mg/L (25 µmol/L)	1.15–11.5 mg/L (25–250 µmol/L)	n/a	[138]
		Saliva	Colorimetric (Griess reagent—citric acid)	(scanner and laptop for signal elaboration)	0.69 mg/L (0.015 µmol/L)	0.04–1 mM (1.8–46 mg/L)	n/a	[36]
		Whole blood	Chemiluminescence (Cu-MOF)	Ring-oven approach for MOF synthesis	0.09 µg/L (2 nmol/L)	0.09–4.6 µg/L (2–100 nmol/L)	n/a	[143]
Detection of Drugs and Illicit Drugs in Biofluids	Alprazolam	Blood; vitreous humor	Colorimetry (silver nanoparticles)	Plastic cassette cabinet (smartphone and laptop for signal elaboration)	smartphone: 10 µg/L (32 nmol/L)	1–10 μg/L (3.2–32 nmol/L)	UV-Vis	[144]
		Urine	Cyclic voltammetry and amperometry	Potentiostat, two-electrode configuration	0.025 µg/L (0.08 nmol/L)	1–300 µg/L (3.2–980 nmol/L)	n/a	[82]
	Diazepam	Urine	Cyclic voltammetry	Potentiostat, two-electrode configuration	0.42 µg/L (1.5 nmol/L)	1 µg/L–1 g/L; (3.5 nmol/L–3.5 mmol/L)	n/a	[83]
	Cathinone mephedrone	Urine	Colorimetry (TMB)	(smartphone and laptop for signal elaboration)	4.34 ng/mL (0.03 μmol/L)	n/a	n/a	[90]
	Cocaine	Urine	Colorimetry (iodine)	(smartphone and laptop for elaborating pictures)	2.3 ng/mL (4.5 µmol/L)	3–15 ng/mL (10–50 µmol/L)	n/a	[64]
		Urine	Colorimetry	Qualitative determination with the naked eye	15 ng/L (50 µmol/L)	n/a	n/a	[145]
		Saliva; blood (rats)	Luminescence (ACA-UCNPs)	(Smartphone)	15.15 µg/L (50 nmol/L) (saliva)	n/a	n/a	[147]
		Oral fluid (spiked)	Surface-enhanced Raman scattering spectroscopy	Raman microscope with 785 nm laser and 1200 line/mm grating	1 ng/mL (3.29 nmol/L)	n/a	n/a	[163]
	Fentanyl	Artificial urine; serum (rat)	Surface-enhanced Raman scattering spectroscopy	Portable Raman spectrometer with a 785 nm excitation laser	0.59 μg/mL (1.75 μmol/L) (urine); 2.78 μg/mL (8.26 μmol/L) (serum)	4–20 μg/mL (12–60 nmol/L)	n/a	[150]
	Ketamine	Saliva	Colorimetry (3,3′,5′,5-tetramethylbenzidine (TMB))	(scanner, smartphone, and laptop for signal elaboration)	0.03 ng/mL (0.1 pmol/L)	1–1000 ng/mL (4.2–4200 pM)	GC-MS	[153]
	MDMA *	Urine; sweat	Differential pulse voltammetry	Potentiostat, two-electrode configuration	19 ng/mL (0.1 µmol/L)	0.19–190 mg/L (1–1000 µmol/L)	n/a	[100]
	Morphine	Saliva	Colorimetry (gold-conjugated anti-immunocomplex Fab)	Piezo-electric inkjet printer (scanner and laptop for signal elaboration)	20 ng/mL (0.07 µmol/L)	20–2000 ng/mL (0.07–7 µmol/L)	n/a	[155]
	Synthetic cannabinoid JWH-073	Saliva	Colorimetry (rhodamine B-loaded polymersomes); Fluorescence	(smartphone and laptop for signal elaboration); fluorescence	0.53 ng/mL (1.6 nmol/L) (sandwich); 0.31 ng/mL (0.9 nmol/L) (competitive); 0.16 ng/mL (0.5 nmol/L) (fluorescence)	5–1000 ng/mL (15–3058 nmol/L)	n/a	[157]
	Tetrahydrocabinol	Saliva	Differential pulse voltammetry	Potentiostat, three-electrode configuration	1.4 µg/L (4.5 nmol/L)	0.01–1.5 mg/L (32–4700 nmol/L)	HPLC-UV/vis	[86]
Detection of Compounds Added to Beverages for Drug-Facilitated Crimes (DFCs)	Flunitrazepam	Carbonated and noncarbonated soft drinks	Potentiometry	Digital ion analyzer	0.17 mg/L (0.55 µmol/L)	0.31 mg/L–3.1 g/L (1 µmol/L–0.01 mol/L)	Titrimetric method	[96]
	GHB *	Beverages	Colorimetric (pentacosadiynoic acid–gabazine)	(Smartphone and laptop for signal elaboration)	9.6 mg/L	n/a	n/a	[158]
	Ketamine	Cola, rum, whiskey	Colorimetric (bromocresol)	(Smartphone and dedicated smartphone app)	2.4 g/L (0.01 mmol/L)	n/a	n/a	[63]
		Beverages	Colorimetric (cobalt thiocyanate); fluorescence (carbon dots–gold nanoparticles); potentiometry	Digital ion analyzer Smartphone and ultraviolet-LED torch with 395 nm light for the fluorimetric and colorimetric detection	10 g/L (42 mmol/L) (colorimetry); 0.048 g/L (0.2 mmol/L) (potentiometry); 0.0008 g/L (0.003 mmol/L) (fluorimetric)	0.04–0.4 mmol/L (colorimetry); 3.2 µmol/L 0.01 mol/L (potentiometry); 0.2–1 mmol/L (fluorimetric)	n/a	[159]
		Alcoholic (whiskeys) and nonalcoholic drinks (real juices)	Cyclic voltammetry	Potentiostat, two-electrode configuration	0.24 µg/L (1 nmol/L)	0.24–1.2 µg/L (1 nmol/L–5 µmol/L)	n/a	[85]
	Metamizole	Whiskey	Square wave voltammetry	Potentiostat, three-electrode configuration	20 mg/L (0.064 mmol/L)	50–250 mg/L (0.16–0.8 mmol/L)	n/a	[77]
	Midazolam				4.8 mg/L (0.015 mmol/L)	25–1000 mg/L (0.077–3.1 mmol/L)	4.8 mg/L (0.015 mmol/L)	
	Scopolamine	Alcohol beverages	Colorimetric (ZnTPP, Methyl orange, Bromocresol green, Iodoplatinate, Dragendorff’s, and Chen’s)	(Scanner and laptop for signal elaboration)	0.6 g/L (2 mmol/L)	n/a	Spectrophotometric method	[161]
	Xylazine	Beverages	Electrochemical	Lab-built portable device	0.06 mg/L (0.27 µmol/L)	0.2 mg/L–0.1 g/L (0.9 µmol/L–0.45 mol/L)	n/a	[84]

* MDMA (methylenedioxymethamphetamine); GHB (gamma-hydroxybutyric acid).

**Table 3:** Combined detection approaches.

Analyte	Matrix	Detection (Sensing Molecule)	Explanation of the Approach	Equipment (Detection System)	Limit of Detection	Linearity	Comparison with a Different Method	Ref.
Cocaine	Seized materials	Cyclic voltammetry–Colorimetry	The device consisted of a first PAD to accommodate the electrodes for EC detection and a second paper layer containing cobalt(II) thiocyanate attached to the electrochemical device for colorimetric detection. A graphite lead modified with an electrochemically grown gold film (graphite–gold) was used as a working electrode to improve electron transfer. Silver tracks were painted with silver ink onto the PAD to create the auxiliary and the reference electrodes. Then, the Ag track was oxidized to AgCl. PCA was also used for sample discrimination.	Potentiostat, three-electrode configuration and smartphone	n/a	n/a	GC-FID	[164]
Fentanyl	Swab from plastic, glass, and metallic surfaces	Surface-enhanced Raman scattering–paper spray mass spectrometry	Commercially available pSERS substrates made by paper inkjet-printed with AgNPs.	Hand-held portable Raman spectrometer with a 785 nm excitation laser	n/a	n/a	n/a	[165]
Cocaine	MeOH	Surface-enhanced Raman scattering–paper spray mass spectrometry	Paper-based SERS substrate made of Whatman grade 1 filter paper immersed in a colloidal suspension of AuNPs.	Raman spectrometer equipped with a 785 nm excitation laser	0.6 ng	n/a	n/a	[149]
Hydrocodone					3.7 ng	n/a	n/a	
JWH-018					26 ng	n/a	n/a	
2C-B					3.8 ng	n/a	n/a	

## 4. Challenges

Although paper-based devices were initially intended as diagnostic tools for use in developing countries, this technology could also offer important advantages when a rapid diagnosis of intoxication is needed, as it occurs in clinical and forensic toxicology [8]. On the other hand, three different aspects require increased attention: (i) readiness of the technology; (ii) fabrication procedures; and (iii) deliverability to the end user.

The first aspect is related to the fact that most of the applications reported in Table 2 are at an initial phase of development. The reported papers are mostly proof-of-concept studies, which often have not achieved a complete validation, including the analysis of a significant number of real samples with a comparison with a reference method. On this basis, the technology readiness level (TRL) of the developed technologies [166] is not homogenous, and it is distributed between TRL 3 and TRL 4 out of nine levels. In fact, for most of the devices, their application has been assessed by laboratory-based experiments finalized at the demonstration of the principle (TRL 3), while few of the devices have also been validated (TRL 4).

The second critical aspect is strictly related to the previous one; in fact, most of the already proposed procedures are often focused on the demonstration of innovative principles, instead of being grounded on well-known reactions providing robust results. An exception is represented by nitrite determination, for which as many as 11 devices have been proposed, and all but one are grounded on the Griess’ reaction (Table 2).

The last aspect concerns the production of devices with an overall quality meeting the needs of the end users. Although some of the proposed devices include information on their durability over time and at different temperature conditions, and this aspect is, in most cases, still to be assessed.

## 5. Perspectives

Although the abovementioned issues represent the major critical points of the proposed devices, two other aspects deserve attention in view of the final developments of paper-based technology. Most of the devices provide only presumptive results because of a general lack of selectivity. Eventually, most of the approaches require the collection of blood or other biofluids and, consequently, the competence of health professionals.

The first problem, selectivity, could be addressed by adopting multiplex devices or including separative strategies. In fact, the identification of the same analyte using different detection principles clearly supports the correctness of identification. This strategy has already been adopted to detect compounds of toxicological interest in biofluids in two reports where colorimetric detection was used in parallel with fluorimetry [157,159]. Other papers using multiplex detection applied this strategy on more simple matrices such as standard solutions or seized products (Table 3). A higher degree of reliability of identification can be achieved by combining surface-enhanced Raman scattering and mass spectrometry which, today, are also available as point-of-need instrumentation [165].

On the other hand, paper-based technologies integrating electrophoretic or chromatographic strategies could highly improve the analytical selectivity because of the separation of the components of the samples before detection. In fact, although the technology of paper electrophoresis was developed during the first decades of the past century [167], different technological aspects have been reconsidered using microchannels with excellent improvements in terms of selectivity. In fact, the available technology allows for the fabrication of channels with diameters down to 1 mm and the use of higher potentials up to 2.5 kV for fast separations (250 s) [28]. On the other hand, chromatography can also be scaled down to microfluidics, with the possibility of achieving analyte separation by tuning the physicochemical proprieties of the support and the mobile phase. However, to the best of our knowledge, no reports on the use of this technology for toxicological purposes are available.

Although most of the paper-based devices integrate all of the steps of the analytical process (sample introduction in the system, derivatization reaction, pH control, analyte oxidation/reduction, and detection), the collection of the biofluid samples is generally performed offline, requiring the manual intervention of health professionals. In order to overcome the complexity of this step, recent advances in paper-based devices have been focused on the fabrication of wearable sensors. Most of these devices integrate flexible surfaces and detection mechanisms suitable for easy and prompt reading of the signals related to the analytical process. In this context, both bi-dimensional and three-dimensional paper devices have been integrated with flexible materials, such as polydimethylsiloxane (PDMS) [168] and poly(3,4-ethylenedioxythiophene):poly(4-styrenesulfonate) (PEDOT:PSS) hydrogel [169,170]. This technology was proposed for the measurements of lithium concentrations in sweat as an alternative procedure to therapeutic drug monitoring in blood. The device integrated a paper surface to collect sweat in a nanostructured potentiometric sensor [171].

## 6. Conclusions

The present review critically describes the main detection methods for paper-based devices and the use of this technology to detect toxicants and compounds of forensic toxicological interest.

At the present moment, although the main challenges are represented by the development of devices with suitable sensitivity and selectivity, the technology readiness level of available devices also remains a crucial aspect that should be taken into consideration. In fact, most proposed devices are proof-of-concept studies showing weak points in terms of the simplicity of implementation in a real context, and they are incomplete or lack appropriate validation studies. Nevertheless, we believe that the low cost and potential effectiveness of these devices in the early diagnostic stage of an intoxication case could provide real benefits for the treatment of patients, offering precious information in the immediacy of the event that otherwise would need complex and time-consuming procedures.

## Figures and Tables

**Table 1:** List of compounds of clinical and forensic toxicological interest for which paper-based devices have been proposed.

Classification on the Basis of the Present Report	Analyte	Category	Effect [3]	Lowest Blood Concentration Causing Toxic Effect (mg/L) [24]
Toxic Compounds	Arsenic	Xenobiotic—Inorganic ion	Arsenic interferes with energy transfer mechanisms	0.05–0.25
	Cyanide	Xenobiotic—Inorganic ion	Cyanide causes cellular hypoxia	0.2–0.5
	Ethanol	Xenobiotic—Small organic molecule	Ethanol acts as a central nervous system depressant	1000–2000
	Nitrite	Xenobiotic—Inorganic ion	Nitrite and derivative compounds (amyl nitrite, butyl nitrite, and isobutyl nitrite) cause methemoglobinemia	32.4 ***
Drugs and Drugs of Abuse	Alprazolam	Licit drug	Alprazolam is used for its anxiolytic action [25]	0.1–0.4
	Diazepam	Licit drug	Diazepam is used as a muscle relaxant or anticonvulsant and for its anti-anxiety action [25]	3–5
	Cathinone. Mephedrone	Illicit drug	Stimulant effect on central and peripheral nervous systems	n/a
	Cocaine	Illicit drug	Cocaine produces a sympathomimetic response	0.25
	Fentanyl	Licit drug	Fentanyl has an antinociceptive activity	0.003 *
	Ketamine	Licit drug	Ketamine is used for its anesthetic action (pediatric medicine)	0.02 (abuse)
	MDMA **	Illicit drug	Stimulant effect on central and peripheral nervous system	0.35–0.50
	Morphine	Illicit drug	Morphine acts as a central nervous system depressant	0.1
	Synthetic Cannabinoids. JWH-073	Illicit drug	Assumption of synthetic cannabinoids results in anxiety, tremulous, and experiencing palpitations	0.002–0.006 ****
	Tetrahydrocannabinol	Illicit drug	It causes sedation, euphoria, and hallucinations	0.34
Psychoactive Substances Added to Beverages for Drug-Facilitated Crimes (DFCs)	Flunitrazepam	Licit drug	Flunitrazepam acts as a hypnotic and anesthetic agent [25]	0.05
	GHB **	Licit drug	GHB causes euphoria, increases sexual desire, and has a CNS depressant effect	80–200
	Ketamine	Licit drug	Ketamine is used for its anesthetic action (pediatric medicine)	0.02 (abuse)
	Metamizole	Licit drug	Metamizole provokes an analgesic effect	20
	Midazolam	Licit drug	Midazolam acts as depressant of the CNS	1–1.5
	Scopolamine	Licit drug	It is illegally used as an incapacitating agent and for inducing submissive behavior in the victim	0.02 (abuse)
	Xylazine	Licit drug	Xylazine is use as a sedative, analgesic, and anesthetic agent (veterinary medicine)	2.3 [25]

* Comatose-fatal blood concentration; ** MDMA (methylenedioxymethamphetamine); GHB (gamma-hydroxybutyric acid); *** average value of nitrite cadaveric blood concentration related to 10 nitrite poisoning cases [26]; **** range value on 85 blood samples which resulted positive for JWH-073 metabolites [27].

## Data Availability

Not applicable.

## Supplementary Materials

The following supporting information can be downloaded at: https://www.mdpi.com/article/10.3390/bios13070743/s1, Table S1: Paper-based devices proposed for clinical and forensic toxicology issues.
