# Supplementary material for: Application of Paper-Based Microfluidic Analytical Devices (µPAD) in Forensic and Clinical Toxicology: A Review
Source: Biosensors (Basel). 2023 Jul 18;13(7):743. doi: 10.3390/bios13070743 (PMC10377625; doi:10.3390/bios13070743)
Supplement: Supplementary file 1 [file biosensors-13-00743-s001.zip › biosensors-2420350-supplementary.pdf]

biosensors

Supplementary Materials

Application of Paper-Based Microfluidic Analytical Devices (μPAD) in Forensic and Clinical Toxicology: A Review

Table S1. Paper-based devices proposed for clinical and forensic toxicology issues.

Category	Analyte	Matrix	Detection [sensing molecule]	Explanation of the approach	Equipment [detection system]	Limit of detection	Linearity	Comparison with a different method	Ref.
Detection of toxic compounds in biofluids	Arsenic	Urine	Colorimetric [AgNPr]	The authors developed and tested an arsenic sensor for detecting As (III) using silver nanoprisms (AgNPr). In particular, it has been observed that As (III) promotes the modification of AgNPr to spherical shape associated to a color change.	Qualitative determination with naked eye	0.5 µg/L (6.7 nmol/L)	0.5 - 1000 µg/L (6.7 - 13000 nmol/L)	Spectrophotometric method (UV-Vis)	[125]
	Cyanide	Liquid samples	Colorimetry [Pd dimethylglioximate]	An homemade device has been designed to detect HCN at the gas phase. The liquid sample is gently insuffled with air, and added with phosphoric acid (30%). The detection was carried out using palladium-dimethylglioximate.	Homemade device integrating air flow module, addition of acid and LED-based detector [LED detector and laptop for signal elaboration]	10 µg/L (0.4 µmol/L)	n/a	GC-MS	[129]
	Cyanide	Blood (plasma)	Colorimetry [Pt complex ([Pt(p-MeC6H4)2(phen)])]	An origami paper-based device constituted by 5 layers is used for i. deposit the sample, ii. separating cyanide from methemoglobin by acidification, iii. collecting the analyte in HCN form, iv. basify HCN to form cyanide, and v. detect the analyte by a	Centrifuge [smartphone and laptop for signal elaboration]	10 µg/L (0.4 µmol/L)	26 - 2600 µg/L (1.0 - 100 µmol/L)	GC-MS	[130]

			color changing from orange to colorless.					
Thiocyanate (metabolite of cyanide)	Urine	Colorimetric [cobalt porphyrin derivative]	The identification of urinary thiocyanate was carried out using a cobalt porphyrin derivative reagent which produced a color changing from pink to yellow	[smartphone and laptop for signal elaboration]	73 µg/L (1.26 µmol/L) [smartphone]; 2.9 mg/L (50 µmol/L) [naked eye]	1 – 100 µmol/L	Ion Chromatography	[131]
Ethanol	Blood (mice blood, sheep blood)	Colorimetric [ABTS]	An enzyme-based reaction is integrated in a gas diffusive device. Ethanol is converted in H2O2 by means of alcohol oxidase. Horseradish peroxidase catalyzes the oxidation of color forming reagent, i.e. 2'-azino-bis (3-ethylbenzothiazoline-6-sulfonic acid) diammonium salt (ABTS) through H2O2	[camera and laptop for signal elaboration]	0.12 g/L (2.6 mmol/L)	0.12 - 1.2 g/L (2.6 - 26 mmol/L)	HS-GC-MS	[132]
	Breath	Colorimetric [Cerium Nanoparticles]	The colorimetric reaction is catalyzed by Cerium nanoparticles (Ce-NPs) which act both as reducing agent and colorimetric reagent. Ce-NPs promotes the conversion of H2O2 to O2 and water. The conversion from CeO2 to Ce2O3 produces a yellow color.	[smartphone and laptop for signal elaboration]	0.01 g/L (0.2 mmol/L)	0.2 - 1.2 g/L (4 - 26 mmol/L)	Electronic Breathalyzer	[162]
Nitrite	Saliva	Colorimetric [Griess reagent]	The authors proposed an electrokinetic stacking process for increasing the	Centrifuge, circuit for generating	75 µg/L (1.6 µmol/L)	75 - 1000 µg/L (1.6 - 21 µmol/L)	n/a	[134]

			sensitivity of the test. After, the concentration of a glass fiber support by means of voltage application, nitrite is then revealed using Griess reagent.	voltage [smartphone]				
	Artificial Urine	Colorimetric [Griess reagent]	Griess reagent used colorimetric reagent for nitrite determination is applied on a paper-based device in which the hydrophobic barriers have been obtained by dissolving adhesive tape using toluene.	[smartphone and laptop for signal elaboration]	2.3 mg/L (50 μmol/L)	N/A	n/a	[135]
	Saliva	Colorimetric [modified Griess reagent -Phosphoric acid]	In order to maximise the color development acetic acid of the Griess' reagent was replaced by 5% H3PO4. Also, the authors separated the two components of the Griess' Reagent, suggesting to use nitrogen to store the device.	Nitrogen to store the device [scanner and laptop for signal elaboration]	0.46 mg/L (10 μmol/L)	0.46 - 46 mg/L (10 - 1000 μmol/L)	Spectrophotometric method (UV-Vis)	[139]
	Saliva	Colorimetric [modified Griess reagent - Phosphoric acid]	The authors tested two different composition of the Griess reagent: i. 116 mM of sulfanilamide; 500 mM of ortho-phosphoric acid; 8 mM of N1NED; ii. 50 mM of sulfanilamide; 330 mM of citric acid; 10 mM of N1NED, and demonstrated the higher sensitivity of reagent i. in detecting nitrite. Authors	[scanner and laptop for signal elaboration]	7.8 μg/L (0.17 μmol/L) [nitrite]; 16.7 mg/L (0.27 mmol/L) [nitrate]	0.23 – 11.5 mg/L (5 - 250 μmol/L) [nitrite]; 9.2 – 55.2 mg/L (0.2 - 1.2 mmol/L) [nitrate]	n/a	[140]

		developed the device also for detecting the main metabolite of nitrite, i.e. nitrate					
Saliva	Colorimetric [Griess reagent - Citric acid]	For the first time the spraying technique has been used for fabricating a single-layered 3D-μPAD, creating three dimensional channels in a single piece of paper	[smartphone, photo box equipped with 84LED and laptop for signal elaboration]	0.46 mg/L (9.6 μmol/L); 3.4 mg/L (74 μmol/L)	0.92 mg/L (0.02 - 5 mmol/L)	n/a	[136]
Saliva	Colorimetric [modified Griess reagent – hydrochloric acid]	A preconcentration step is proposed for detecting nitrite in saliva specimens using stamped μPADs, multiple deposition of the sample (n=10) allows to increase the sensitivity of the detection.	[scanner and laptop for signal elaboration]	0.26 mg/L (5.6 μmol/L) (it is not specified if the LOD is calculated using standard solution or saliva specimens)	0.26 – 1.15 mg/L (5.6 - 25 mmol/L)	Spectrophotometric method	[141]
Saliva (artificial)	Colorimetric [modified Griess reagent – hydrochloric acid]	A computer simulation has been carried out for designing a paper-based micromixer device: as a result of the geometry device, sample and sulfanilamide were mixed before the interaction with NED (spotted on opposite end of the device).	[smartphone and laptop for signal elaboration]	0.26 mg/L (5.7 μmol/L)	0.26 - 46 mg/L (5.7 – 1000 μmol/L)	HPLC-UV	[142]
Urine / Serum	Colorimetric [Griess reagent] citric acid	The determination of nitrite ion was carried out Griess reagent integrated in a	[smartphone and laptop for	0.2 mg/L	0.23 - 27.6 mg/L	n/a	[137]

				bidimensional device (2D-µPAD) for detecting the analyte in urine, and in a three-dimensional device (3D-µPAD) for identifying nitrite ion in serum.	signal elaboration]	(4.3 µmol/L) [serum]; 0.1 mg/L (2.3 µmol/L) [urine]	(5 - 600 µmol/L) [serum]; 0.23 - 4.6 mg/L (5 - 100 µmol/L) [urine]		
		Saliva	Colorimetric [Griess reagent] citric acid	The quantification of saliva nitrite was carried out by means of Griess reagent integrated in a deepstick-like paper-based device	[smartphone and laptop for signal elaboration]	1.15 mg/L (25 µmol/L)	1.15 - 11.5 mg/L (25 - 250 µmol/L)	n/a	[138]
		Saliva	Colorimetric [Griess reagent - Citric acid]	A custom-made extruder allows to fabricate a device in single step consisting only in the deposition of the wax on the paper. In fact, the rapid solidification of the printed wax pattern does not require to melt wax	[scanner and laptop for signal elaboration]	0.69 mg/L (0.015 µmol/L)	0.04 - 1 mM (1.8 - 46 mg/L)	n/a	[36]
		Whole Blood	Chemiluminescence [Cu-MOF]	The authors proposed the use of a Cu-MOF system for catalyzing nitrite-peroxide hydrogen reaction	Ring-oven approach for MOF synthesis	0.09 µg/L (2 nmol/L)	0.09 – 4.6 µg/L (2 – 100 nmol/L)	n/a	[143]
Detection of drugs and illicit drugs in biofluids	Alprazolam	Blood; Vitreous Humor	Colorimetry [Silver nanoparticles]	The detection of alprazolam was performed by evaluating the color changing due to the aggregation of silver nanoparticles (AgNPs). The aggregation is promoted by the formation of H-bonding between alprazolam aminogroup and NP	Plastic cassette cabinet [smartphone and laptop for signal elaboration]	smartphone: 10 µg/L (32 nmol/L)	1 – 10 µg/L (3.2 – 32 nmol/L)	UV-Vis	[144]

			stabilizing (citrate) carboxylic groups.					
	Urine	Cyclic Voltammetry and amperometry	Working (WE) and counter electrode (CE) were firstly assembled on the paper using commercially available conducting carbon ink. Then, methylene blue doped silver core palladium (Ag@ Pd) shell nano-hybrids were drop casted on the carbon-based WE followed by room temperature air drying. Alprazolam was reduced to corresponding dihydro derivative on the methylene blue Ag@Pd modified surface.	Potentiostat, two electrode configuration	0.025 µg/L (0.08 nmol/L)	1 – 300 µg/L (3.2 - 980 nmol/L)	n/a	[82]
Diazepam	Urine	Cyclic Voltammetry	Silica coated gold nanorods (Si@GNRs) were synthesized and physically deposited by drop casting on the working electrode previously fabricated on paper using carbon ink. A reduction peak was observed due to the reduction of azomethine functional group at the position four of the benzodiazepine ring.	Potentiostat, two electrode configuration.	0.42 µg/L (1.5 nmol/L)	1 µg/L - 1 g/L; (3.5 nmol/L - 3.5 mmol/L)	n/a	[83]
Cathinone. Mephedrone	Urine	Colorimetry [TMB]	The device was based on a competitive immunoassay using an anti-cathinone	[smartphone and laptop for	4.34 ng/mL (0.03 µmol/L)	n/a	n/a	[90]

			antibody and cathinone-HRP . The sample adsorbed on the paper is washed twice with water, if the sample contained the analyte, the color development promoted by 3,3',5,5′-tetramethylbenzidine (TMB) is proportionally reduced.	signal elaboration]				
Cocaine	Urine	Colorimetry [iodine]	The detection of the analyte was carried out by using an aptamer-crosslinked glucoamylase-trapped hydrogel integrated in a paper-based device. In particular, the structure of hydrogel is broken as result of the interaction between the target compound, i.e., and aptamers which stabilize the hydrogel, and it releases glucoamylase. The enzyme then catalyzes glucose production, which fluids on paper where reacts with glucose oxidase producing hydrogen peroxide. The reaction product is then reduced by horseradish peroxidase, while iodide (colorless) is oxidated to iodine (brown).	[smartphone and Laptop for elaborating pictures]	2.3 ng/mL (4.5 µmol/L)	3 - 15 ng/mL (10 - 500 µmol/L)	n/a	[64]

	Urine	Colorimetry	The authors proposed a device based on the formation of a hydrogel. In particular, the presence of the analyte does not permit the formation of the hydrogel, because of the primary interaction with aptamers, which act as structure stabilizers. The formation of the hydrogel does not allow the solution to fluid towards the colorimetric reagent area. The principle was demonstrated by proposing a distance-based approach and a device based on the formation of the color.	Qualitative determination with naked eye	15 ng/L (50 µmol/L)	n/a	n/a	[145]
	Saliva; Blood (rats)	Luminescence [ACA-UCNPs]	Up-conversion nanoparticles (UCNPs) functionalized with anticocaine aptamers. In the presence of cocaine, the UCNPs luminescence is quenched. The luminescence change can be observed by naked eye for qualification or recorded by a smartphone camera.	[smartphone]	15.15 µg/L (50 nmol/L) [saliva]	n/a	n/a	[147]
	oral fluid (spiked)	Surface Enhanced Raman Scattering Spectroscopy	A paper-based SERS substrate is produced by depositing nano gold (from an HAuCl4 precursor solution) on paper using the	Raman microscope with 785 nm laser and 1200 line/mm grating	1 ng/mL (3.29 nmol/L)	n/a	n/a	[163]

			plasma-assisted chemical vapor deposition.					
Fentanyl	artificial urine; serum (rat)	Surface Enhanced Raman Scattering Spectroscopy	A liquid/liquid self-assembled film of gold nanospheres was deposited on filter paper. The substrate was immersed in NaCl (0.5 M) solution to improve its sensitivity.	Portable Raman spectrometer with a 785 nm excitation laser	0.59 µg/mL (1.75 µmol/L) [urine]; 2.78 µg/mL (8.26 µmol/L) [serum]	4–20 µg/mL (12 - 60 nmol/L)	n/a	[150]
Ketamine	Saliva	Colorimetry [3,3′,5′,5 -tetramethylbenzidine (TMB)]	A competitive ELISA test was integrated in a µPAD. Detection of ketamine utilizes ketamine antibody (K-Ab) and ketamine conjugated horseradish peroxidase, with 3,3′,5′,5-tetramethylbenzidine (TMB) which is used as chromogenic substrate.	[scanner, smartphone and laptop for signal elaboration]	0.03 ng/mL (0.1 pmol/L)	1 - 1000 ng/mL (4.2 - 4200 pM)	GC-MS	[153]
MDMA*	Urine, sweat	Differential Pulse Voltammetry	A two electrode circuit consisting of a counter electrode (CE) and a working electrode (WE) was created on paper using carbon ink. The working electrode was then coated with zinc oxide nanorods (ZnONRs) by drop casting to promote electro-oxidation of MDMA at the electrode surface.	Potentiostat, two electrode configuration	19 ng/mL (0.1 µmol/L)	0.19 – 190 mg/L (1 - 1000 µmol/L)	n/a	[100]
Morphine	Saliva	Colorimetry [gold-conjugated anti-immunocomplex Fab]	A paper-based later-flow assay for detecting morphine in saliva specimens was	piezo-electric inkjet printer [scanner and	20 ng/mL (0.07 µmol/L)	20 - 2000 ng/mL (0.07 – 7 µmol/L)	n/a	[155]

				developed. The two reagents, anti-morphine Fab M1 and anti-human F(ab′)2, were printed on the paper to form test and control lines.	laptop for signal elaboration]				
	Synthetic Cannabinoid. JWH-073	Saliva	Colorimetry [rhodamine B–loaded polymersomes] - Fluorescence	A paper-based later-flow assay system was developed for detecting a synthetic cannabinoid (JWH-073) in saliva specimens. The authors proposed both competitive and sandwich strategies. The system used a rhodamine B–loaded polymersome functionalized with anti-K2 antibodies.	[smartphone and laptop for signal elaboration] - also fluorescence	0.53 ng/mL (1.6 nmol/L) [sandwich]; 0.31 ng/mL (0.9 nmol/L) [competitive]; 0.16 ng/mL (0.5 nmol/L) [fluorescence]	5 - 1000 ng/mL (15 - 3058 nmol/L)	n/a	[157]
	Tetrahydrocabinol	Saliva	Differential Pulse Voltammetry	The device enables the detection of Δ^9-THC as effective biomarkers in saliva. It consists of a circular PAD for sample loading and pre-treatment attached to a PVC layer containing the three electrodes circuit assembled by screen printing. A graphene ink containing 3% of copper-phthalocyanine was used to create the WE while Ag/AgCl ink was used for the reference electrode.	Potentiostat, three electrode configuration	1.4 µg/L (4.5 nmol/L)	0.01 - 1.5 mg/L (32 - 4700 nmol/L)	HPLC-UV/vis	[86]
Detection of compoun	Flunitrazepam	Carbonated and non-	Potentiometry	All solid-state miniaturized ion-sensing devices with reference and indicator	Digital Ion Analyzer	0.17 mg/L (0.55 µmol/L)	0.31 mg/L – 3.1 g/L (1 µmol/L - 0.01 mol/L)	Titrimetric method	[96]

ds added to beverages for drug-facilitated crimes(DFC)		carbonated soft drinks		electrodes were screen-printed on a paper substrate using silver and carbon inks, respectively. PEDT nanoparticle dispersion was applied on carbon surface and the sensor was used for determination of flunitrazepam					
	GHB*	Beverages	Colorimetric [pentacosadiynoic acid-gabazine]	The proposed device was based on the interaction between the analyte and pentacosadiynoic acid-gabazine reagent which turns from blu to red	[smartphone and laptop for signal elaboration]	9.6 mg/L	n/a	n/a	[158]
	Ketamine	Cola, Rum, Whiskey	Colorimetric [bromocresol]	A distance-based device using bromocresol has been designed for detecting ketamine in different kind of beverages	[smartphone and dedicated smartphone app]	2.4 g/L (0.01 mmol/L)	n/a	n/a	[63]
		Beverages	Colorimetric [cobalt thiocyanate]; Fluorescence [Carbon dots-gold nanoparticles]; Potentiometry	A Paper-based device was designed to combine potentiometric, fluorimetric and colorimetric detection. It consists of a first paper layer where two electrodes are printed using MWCNTs ink for further application of a PANI dispersion and PVC indicator and reference membranes. A second PAD with three zones corresponding to detection	Digital ion analyzer Smartphone and ultraviolet-LED torch with 395 nm light for the fluorimetric and colorimetric detection	10 g/L (42 mmol/L) [colorimetry]; 0.048 g/L (0.2 mmol/L) [potentiometry]; 0.0008 g/L (0.003 mmol/L) [fluorimetric]	0.04 - 0.4 mmol/L [colorimetry]; 3.2 μmol/L 0.01 mol/L [potentiometry]; 0.2 - 1 mmol/L [fluorimetric]	n/a	[159]

			mechanism was aligned. Carbon dots-gold nanoparticles and cobalt thiocyanate were used in the fluorescence and color detection zones, respectively.					
	Alcoholic (whiskeys) and non-alcoholic drinks (real juices)	Cyclic Voltammetry	Graphene-oxide nanocrystals (Zeo–GO) were deposited on the surface of the working electrode previously fabricated on paper using carbon ink and then kept at room temperature for drying. The redox transition of the methylene blue (MB) was exploited for the determination of ketamine drug.	Potentiostat, two electrode configuration.	0.24 µg/L (1 nmol/L)	0.24 - 1.2 µg/L (1 nmol/L – 5 µmol/L)	n/a	[85]
Metamizole	Whiskey	Square Wave Voltammetry	A three electrodes circuit was hand drawn on vegetal paper with a graphite pencil and then the reference electrode (RE) was painted with silver ink.	Potentiostat, three electrode configuration	20 mg/L (0.064 mmol/L)	50 – 250 mg/L (0.16 - 0.8 mmol/L)	n/a	[77]
Midazolam					4.8 mg/L (0.015 mmol/L)	25 – 1000 mg/L (0.077 - 3.1 mmol/L)	4.8 mg/L (0.015 mmol/L)	
Scopolamine	Alcohol beverages	Colorimetric [ZnTPP, Methyl orange, Bromocresol green, Iodoplatinate, Dragendorff's, Chen's	A multiplex device based on six different colorimetric reagents was used for identifying eight psychoactive substances (scopolamine, atropine, cocaine, morphine, ephedrine, caffeine, dipyrone,	[scanner and laptop for signal elaboration]	0.6 g/L (2 mmol/L)	n/a	Spectrophotometric method	[161]

			and alprazolam). A chemometric approach was implemented and used for detecting scopolamine in four alcoholic beverages					
Xylazine	Beverage	Electrochemical	A three-electrode system consisting of a WE, a (RE) and a CE was deposited on paper using a graphene ink. Ag/AgCl ink was applied with a paintbrush to the RE. Polyaniline modification of WE provide an electron transfer medium with a larger effective surface area that promoted charge transfer.	Lab-built portable device	0.06 mg/L (0.27 µmol/L)	0.2 mg/L-0.1 g/L (0.9 µmol/L – 0.45 mol/L)	n/a	[84]

*MDMA (Methylenedioxymethamphetamine); GHB (gamma-hydroxybutyric acid).

References

1. Paracelso (Philippus Aureolus Theophrastus Bombastus von Hohenheim). *Opera Omnia Medico-Chemico-Chirurgica, Tribus Voluminibus Comprehensa*.
2. Negrusz, A.; Cooper, G. *Clarke's Analytical Forensic Toxicology*; Pharmaceutical Press: London, UK, 2013; ISBN 978-0-85711-054-1.
3. Riafi, N.; Horvath, A.R.; Wittwer, C.T. *Tietz Textbook of Clinical Chemistry and Molecular Diagnostics*; 6th ed.; Elsevier: Amsterdam, The Netherlands, 2017; ISBN 978-0-323-35921-4.
4. Convery, N.; Gadegaard, N. 30 Years of Microfluidics. *Micro Nano Eng.* **2019**, *2*, 76–91. https://doi.org/10.1016/j.mne.2019.01.003.
5. Performance Evaluation of Handheld Raman Spectroscopy for Cocaine Detection in Forensic Case Samples. Kranenburg. 2021. Drug Testing and Analysis. Wiley Online Library. Available online: https://analyticalsciencejournals.onlinelibrary.wiley.com/doi/10.1002/dta.2993 (accessed on 14 June 2023).
6. A Calibration Friendly Approach to Identify Drugs of Abuse Mixtures with a Portable Near-infrared Analyzer—Kranenburg. 2022. Drug Testing and Analysis. Wiley Online Library. Available online: https://analyticalsciencejournals.onlinelibrary.wiley.com/doi/10.1002/dta.3231 (accessed on 14 June 2023).
7. Paixão, T.R.; Coltro, W.K.T.; Salles, M.O. *Forensic Analytical Methods*; Royal Society of Chemistry: London, UK, 2019.
8. Noviana, E.; Ozer, T.; Carrell, C.S.; Link, J.S.; McMahon, C.; Jang, I.; Henry, C.S. Microfluidic Paper-Based Analytical Devices: From Design to Applications. *Chem. Rev.* **2021**, *121*, 11835–11885. https://doi.org/10.1021/acs.chemrev.0c01335.
9. Müller, R.H.; Clegg, D.L. Automatic Paper Chromatography. *Anal. Chem.* **1949**, *21*, 1123–1125. https://doi.org/10.1021/ac60033a032.
10. Martinez, A.W.; Phillips, S.T.; Butte, M.J.; Whitesides, G.M. Patterned Paper as a Platform for Inexpensive, Low Volume, Portable Bioassays. *Angew. Chem.* **2007**, *46*, 1318–1320. https://doi.org/10.1002/anie.200603817.
11. Kung, C.-T.; Hou, C.-Y.; Wang, Y.-N.; Fu, L.-M. Microfluidic Paper-Based Analytical Devices for Environmental Analysis of Soil, Air, Ecology and River Water. *Sens. Actuators B Chem.* **2019**, *301*, 126855. https://doi.org/10.1016/j.snb.2019.126855.
12. Mahato, K.; Srivastava, A.; Chandra, P. Paper Based Diagnostics for Personalized Health Care: Emerging Technologies and Commercial Aspects. *Biosens. Bioelectron.* **2017**, *96*, 246–259. https://doi.org/10.1016/j.bios.2017.05.001.
13. Musile, G.; Agard, Y.; Wang, L.; De Palo, E.F.; McCord, B.; Tagliaro, F. Paper-Based Microfluidic Devices: On-Site Tools for Crime Scene Investigation. *TrAC Trends Anal. Chem.* **2021**, *143*, 116406. https://doi.org/10.1016/j.trac.2021.116406.
14. Ray, R.; Prabhu, A.; Prasad, D.; Garlapati, V.K.; Aminabhavi, T.M.; Mani, N.K.; Simal-Gandara, J. Paper-Based Microfluidic Devices for Food Adulterants: Cost-Effective Technological Monitoring Systems. *Food Chem.* **2022**, *390*, 133173. https://doi.org/10.1016/j.foodchem.2022.133173.
15. Noviana, E.; Carrão, D.B.; Pratiwi, R.; Henry, C.S. Emerging Applications of Paper-Based Analytical Devices for Drug Analysis: A Review. *Anal. Chim. Acta* **2020**, *1116*, 70–90. https://doi.org/10.1016/j.aca.2020.03.013.
16. Land, K.J.; Boeras, D.I.; Chen, X.-S.; Ramsay, A.R.; Peeling, R.W. Reassured Diagnostics to Inform Disease Control Strategies, Strengthen Health Systems and Improve Patient Outcomes. *Nat. Microbiol.* **2019**, *4*, 46–54. https://doi.org/10.1038/s41564-018-0295-3.
17. Lu, J.; Ge, S.; Ge, L.; Yan, M.; Yu, J. Electrochemical DNA Sensor Based on Three-Dimensional Folding Paper Device for Specific and Sensitive Point-of-Care Testing. *Electrochim. Acta* **2012**, *80*, 334–341. https://doi.org/10.1016/j.electacta.2012.07.024.
18. Musile, G.; Agard, Y.; De Palo, E.F.; Shestakova, K.; Bortolotti, F.; Tagliaro, F. Thanatochemistry at the Crime Scene: A Microfluidic Paper-Based Device for Ammonium Analysis in the Vitreous Humor. *Anal. Chim. Acta* **2019**, *1083*, 150–156. https://doi.org/10.1016/j.aca.2019.07.033.
19. Peters, K.L.; Corbin, I.; Kaufman, L.M.; Zreibe, K.; Blanes, L.; McCord, B.R. Simultaneous Colorimetric Detection of Improvised Explosive Compounds Using Microfluidic Paper-Based Analytical Devices (MPADs). *Anal. Methods* **2015**, *7*, 63–70. https://doi.org/10.1039/c4ay01677g.
20. Cromartie, R.L.; Wardlow, A.; Duncan, G.; McCord, B.R. Development of a Microfluidic Device (MPADs) for Forensic Serological Analysis. *Anal. Methods* **2019**, *11*, 587–595. https://doi.org/10.1039/C8AY02080A.
21. Musile, G.; Agard, Y.; Pesavento, S.; De Palo, E.F.; Dorizzi, R.M.; Bortolotti, F. An Origami Microfluidic Paper Device for On-Site Assessment of Urine Tampering. First Use of Nessler's Reagent for the Colorimetric Determination of Creatinine. *Anal. Chim. Acta* **2023**, *1237*, 340610. https://doi.org/10.1016/j.aca.2022.340610.
22. Musile, G.; Wang, L.; Bottoms, J.; Tagliaro, F.; McCord, B. The Development of Paper Microfluidic Devices for Presumptive Drug Detection. *Anal. Methods* **2015**, *7*, 8025–8033. https://doi.org/10.1039/C5AY01432H.
23. Silva, T.G.; de Araujo, W.R.; Muñoz, R.A.A.; Richter, E.M.; Santana, M.H.P.; Coltro, W.K.T.; Paixão, T.R.L.C. Simple and Sensitive Paper-Based Device Coupling Electrochemical Sample Pretreatment and Colorimetric Detection. *Anal. Chem.* **2016**, *88*, 5145–5151. https://doi.org/10.1021/acs.analchem.6b00072.
24. Schulz, M.; Schmoldt, A.; Andresen-Streichert, H.; Iwersen-Bergmann, S. Revisited: Therapeutic and Toxic Blood Concentrations of More than 1100 Drugs and Other Xenobiotics. *Crit. Care* **2020**, *24*, 195. https://doi.org/10.1186/s13054-020-02915-5.
25. Baselt, R. *Disposition of Toxic Drugs and Chemicals in Man*; 11th ed.; Biomedical Publications: Seal Beach, CA, USA, 2017. ISBN 978-0-692-77499-1.
26. Kim, M.; Kim, S.; Yang, W.; Sim, J. Determination of Nitrite and Nitrate in Postmortem Whole Blood Samples of 10 Sodium Nitrite Poisoning Cases: The Importance of Nitrate in Determining Nitrite Poisoning. *Forensic Sci. Int.* **2022**, *335*, 111279.

https://doi.org/10.1016/j.forsciint.2022.111279.
27. Ozturk, S.; Ozturk, Y.E.; Yeter, O.; Alpertunga, B. Application of a Validated LC–MS/MS Method for JWH-073 and Its Metabolites in Blood and Urine in Real Forensic Cases. *Forensic Sci. Int.* **2015**, *257*, 165–171. https://doi.org/10.1016/j.forsciint.2015.08.013.
28. de Araujo, W.R.; Paixão, T.R.L.C. *Paper-Based Analytical Devices for Chemical Analysis and Diagnostics*; 1st ed.; Elsevier: Amsterdam, The Netherlands, 2021; ISBN 978-0-12-820534-1.
29. Araujo, W.R.d.; Paixão, T.R.L.C. Fabrication of Disposable Electrochemical Devices Using Silver Ink and Office Paper. *Analyst* **2014**, *139*, 2742–2747. https://doi.org/10.1039/C4AN00097H.
30. Pelton, R. Bioactive Paper Provides a Low-Cost Platform for Diagnostics. *TrAC Trends Anal. Chem.* **2009**, *28*, 925–942. https://doi.org/10.1016/j.trac.2009.05.005.
31. de Araujo, W.R.; Frasson, C.M.R.; Ameku, W.A.; Silva, J.R.; Angnes, L.; Paixão, T.R.L.C. Single-Step Reagentless Laser Scribing Fabrication of Electrochemical Paper-Based Analytical Devices. *Angew. Chem.* **2017**, *56*, 15113–15117. https://doi.org/10.1002/anie.201708527.
32. Fridley, G.E.; Holstein, C.A.; Oza, S.B.; Yager, P. The Evolution of Nitrocellulose as a Material for Bioassays. *MRS Bull.* **2013**, *38*, 326–330. https://doi.org/10.1557/mrs.2013.60.
33. Li, X.; Tian, J.; Nguyen, T.; Shen, W. Paper-Based Microfluidic Devices by Plasma Treatment. *Anal. Chem.* **2008**, *80*, 9131–9134. https://doi.org/10.1021/ac801729t.
34. Lu, Y.; Shi, W.; Jiang, L.; Qin, J.; Lin, B. Rapid Prototyping of Paper-Based Microfluidics with Wax for Low-Cost, Portable Bioassay. *Electrophoresis* **2009**, *30*, 1497–1500. https://doi.org/10.1002/elps.200800563.
35. Carrilho, E.; Martinez, A.W.; Whitesides, G.M. Understanding Wax Printing: A Simple Micropatterning Process for Paper-Based Microfluidics. *Anal. Chem.* **2009**, *81*, 7091–7095. https://doi.org/10.1021/ac901071p.
36. Chiang, C.-K.; Kurniawan, A.; Kao, C.-Y.; Wang, M.-J. Single Step and Mask-Free 3D Wax Printing of Microfluidic Paper-Based Analytical Devices for Glucose and Nitrite Assays. *Talanta* **2019**, *194*, 837–845. https://doi.org/10.1016/j.talanta.2018.10.104.
37. Le, S.; Zhou, H.; Nie, J.; Cao, C.; Yang, J.; Pan, H.; Li, J.; Zhang, Y. Fabrication of Paper Devices via Laser-Heating-Wax-Printing for High-Tech Enzyme-Linked Immunosorbent Assays with Low-Tech Pen-Type PH Meter Readout. *Analyst* **2017**, *142*, 511–516. https://doi.org/10.1039/C6AN02422J.
38. Olkkonen, J.; Lehtinen, K.; Erho, T. Flexographically Printed Fluidic Structures in Paper. *Anal. Chem.* **2010**, *82*, 10246–10250. https://doi.org/10.1021/ac1027066.
39. Abe, K.; Suzuki, K.; Citterio, D. Inkjet-Printed Microfluidic Multianalyte Chemical Sensing Paper. *Anal. Chem.* **2008**, *80*, 6928–6934. https://doi.org/10.1021/ac800604v.
40. Maejima, K.; Tomikawa, S.; Suzuki, K.; Citterio, D. Inkjet Printing: An Integrated and Green Chemical Approach to Microfluidic Paper-Based Analytical Devices. *RSC Adv.* **2013**, *3*, 9258–9263. https://doi.org/10.1039/C3RA40828K.
41. Wang, J.; Monton, M.R.N.; Zhang, X.; Filipe, C.D.M.; Pelton, R.; Brennan, J.D. Hydrophobic Sol-Gel Channel Patterning Strategies for Paper-Based Microfluidics. *Lab Chip* **2014**, *14*, 691–695. https://doi.org/10.1039/c3lc51313k.
42. Ghosh, R.; Gopalakrishnan, S.; Savitha, R.; Renganathan, T.; Pushpavanam, S. Fabrication of Laser Printed Microfluidic Paper-Based Analytical Devices (LP-MPADs) for Point-of-Care Applications. *Sci. Rep.* **2019**, *9*, 7896. https://doi.org/10.1038/s41598-019-44455-1.
43. Gallibu, C.; Gallibu, C.; Avoundjian, A.; Gomez, F.A. Easily Fabricated Microfluidic Devices Using Permanent Marker Inks for Enzyme Assays. *Micromachines* **2016**, *7*, 6. https://doi.org/10.3390/mi7010006.
44. Ostad, M.A.; Hajinia, A.; Heidari, T. A Novel Direct and Cost Effective Method for Fabricating Paper-Based Microfluidic Device by Commercial Eye Pencil and Its Application for Determining Simultaneous Calcium and Magnesium. *Microchem. J.* **2017**, *133*, 545–550. https://doi.org/10.1016/j.microc.2017.04.031.
45. Sousa, L.R.; Duarte, L.C.; Coltro, W.K.T. Instrument-Free Fabrication of Microfluidic Paper-Based Analytical Devices through 3D Pen Drawing. *Sens. Actuators B Chem.* **2020**, *312*, 128018. https://doi.org/10.1016/j.snb.2020.128018.
46. Nie, J.; Zhang, Y.; Lin, L.; Zhou, C.; Li, S.; Zhang, L.; Li, J. Low-Cost Fabrication of Paper-Based Microfluidic Devices by One-Step Plotting. *Anal. Chem.* **2012**, *84*, 6331–6335. https://doi.org/10.1021/ac203496c.
47. Songjaroen, T.; Dungchai, W.; Chailapakul, O.; Laiwattanapaisal, W.; Novel, Simple and Low-Cost Alternative Method for Fabrication of Paper-Based Microfluidics by Wax Dipping. *Talanta* **2011**, *85*, 2587–2593. https://doi.org/10.1016/j.talanta.2011.08.024.
48. Cardoso, T.M.G.; de Souza, F.R.; Garcia, P.T.; Rabelo, D.; Henry, C.S.; Coltro, W.K.T. Versatile Fabrication of Paper-Based Microfluidic Devices with High Chemical Resistance Using Scholar Glue and Magnetic Masks. *Anal. Chim. Acta* **2017**, *974*, 63–68. https://doi.org/10.1016/j.aca.2017.03.043.
49. Chitnis, G.; Ding, Z.; Chang, C.-L.; Savran, C.A.; Ziaie, B. Laser-Treated Hydrophobic Paper: An Inexpensive Microfluidic Platform. *Lab Chip* **2011**, *11*, 1161–1165. https://doi.org/10.1039/C0LC00512F.
50. Curto, V.F.; Lopez-Ruiz, N.; Capitan-Vallvey, L.F.; Palma, A.J.; Benito-Lopez, F.; Diamond, D. Fast Prototyping of Paper-Based Microfluidic Devices by Contact Stamping Using Indelible Ink. *RSC Adv.* **2013**, *3*, 18811–18816. https://doi.org/10.1039/C3RA43825B.
51. de Freitas, S.V.; de Souza, F.R.; Rodrigues Neto, J.C.; Vasconcelos, G.A.; Abdelnur, P.V.; Vaz, B.G.; Henry, C.S.; Coltro, W.K.T. Uncovering the Formation of Color Gradients for Glucose Colorimetric Assays on Microfluidic Paper-Based Analytical Devices by Mass Spectrometry Imaging. *Anal. Chem.* **2018**, *90*, 11949–11954. https://doi.org/10.1021/acs.analchem.8b02384.

52. Zhao, X.; Zhao, H.; Yan, L.; Li, N.; Shi, J.; Jiang, C. Recent Developments in Detection Using Noble Metal Nanoparticles. *Crit. Rev. Anal. Chem.* **2020**, *50*, 97–110. https://doi.org/10.1080/10408347.2019.1576496.
53. Yamada, K.; Takaki, S.; Komuro, N.; Suzuki, K.; Citterio, D. An Antibody-Free Microfluidic Paper-Based Analytical Device for the Determination of Tear Fluid Lactoferrin by Fluorescence Sensitization of Tb3+. *Analyst* **2014**, *139*, 1637–1643. https://doi.org/10.1039/C3AN01926H.
54. De Tarso Garcia, P.; Garcia Cardoso, T.M.; Garcia, C.D.; Carrilho, E.; Tomazelli Coltro, W.K. A Handheld Stamping Process to Fabricate Microfluidic Paper-Based Analytical Devices with Chemically Modified Surface for Clinical Assays. *RSC Adv.* **2014**, *4*, 37637–37644. https://doi.org/10.1039/c4ra07112c.
55. Evans, E.; Moreira Gabriel, E.F.; Benavidez, T.E.; Tomazelli Coltro, W.K.; Garcia, C.D. Modification of Microfluidic Paper-Based Devices with Silica Nanoparticles. *Analyst* **2014**, *139*, 5560–5567. https://doi.org/10.1039/C4AN01147C.
56. Figueredo, F.; Garcia, P.T.; Cortón, E.; Coltro, W.K.T. Enhanced Analytical Performance of Paper Microfluidic Devices by Using Fe_3O_4 Nanoparticles, MWCNT, and Graphene Oxide. *ACS Appl. Mater. Interfaces* **2016**, *8*, 11–15. https://doi.org/10.1021/acsami.5b10027.
57. Jiang, C.; Liu, B.; Han, M.-Y.; Zhang, Z. Fluorescent Nanomaterials for Color-Multiplexing Test Papers toward Qualitative/Quantitative Assays. *Small Methods* **2018**, *2*, 1700379. https://doi.org/10.1002/smtd.201700379.
58. Gabriel, E.F.M.; Garcia, P.T.; Cardoso, T.M.G.; Lopes, F.M.; Martins, F.T.; Coltro, W.K.T. Highly Sensitive Colorimetric Detection of Glucose and Uric Acid in Biological Fluids Using Chitosan-Modified Paper Microfluidic Devices. *Analyst* **2016**, *141*, 4749–4756. https://doi.org/10.1039/C6AN00430J.
59. Evans, E.; Gabriel, E.F.M.; Coltro, W.K.T.; Garcia, C.D. Rational Selection of Substrates to Improve Color Intensity and Uniformity on Microfluidic Paper-Based Analytical Devices. *Analyst* **2014**, *139*, 2127–2132. https://doi.org/10.1039/C4AN00230J.
60. Martinez, A.W.; Phillips, S.T.; Carrilho, E.; Thomas, S.W.; Sindi, H.; Whitesides, G.M. Simple Telemedicine for Developing Regions: Camera Phones and Paper-Based Microfluidic Devices for Real-Time, Off-Site Diagnosis. *Anal. Chem.* **2008**, *80*, 3699–3707. https://doi.org/10.1021/ac800112r.
61. Morbioli, G.G.; Mazzu-Nascimento, T.; Milan, L.A.; Stockton, A.M.; Carrilho, E. Improving Sample Distribution Homogeneity in Three-Dimensional Microfluidic Paper-Based Analytical Devices by Rational Device Design. *Anal. Chem.* **2017**, *89*, 4786–4792. https://doi.org/10.1021/acs.analchem.6b04953.
62. Kaneta, T.; Alahmad, W.; Varanusupakul, P. Microfluidic Paper-Based Analytical Devices with Instrument-Free Detection and Miniaturized Portable Detectors. *Appl. Spectrosc. Rev.* **2019**, *54*, 117–141. https://doi.org/10.1080/05704928.2018.1457045.
63. Narang, J.; Singhal, C.; Mathur, A.; Dubey, A.K.; Krishna, A.; Anil, A.; Pundir, C.S. Naked-Eye Quantitative Assay on Paper Device for Date Rape Drug Sensing via Smart Phone APP. *Vacuum* **2018**, *153*, 300–305. https://doi.org/10.1016/j.vacuum.2018.03.056.
64. Tian, T.; An, Y.; Wu, Y.; Song, Y.; Zhu, Z.; Yang, C. Integrated Distance-Based Origami Paper Analytical Device for One-Step Visualized Analysis. *ACS Appl. Mater. Interfaces* **2017**, *9*, 30480–30487. https://doi.org/10.1021/acsami.7b09717.
65. Shen, L.; Hagen, J.A.; Papautsky, I. Point-of-Care Colorimetric Detection with a Smartphone. *Lab Chip* **2012**, *12*, 4240–4243. https://doi.org/10.1039/C2LC40741H.
66. Ye, X.; Zhang, F.; Yang, L.; Yang, W.; Zhang, L.; Wang, Z. Paper-Based Multicolor Sensor for on-Site Quantitative Detection of 2,4-Dichlorophenoxyacetic Acid Based on Alkaline Phosphatase-Mediated Gold Nanobipyramids Growth and Colorimeter-Assisted Method for Quantifying Color. *Talanta* **2022**, *245*, 123489. https://doi.org/10.1016/j.talanta.2022.123489.
67. Lopez-Ruiz, N.; Curto, V.F.; Erenas, M.M.; Benito-Lopez, F.; Diamond, D.; Palma, A.J.; Capitan-Vallvey, L.F. Smartphone-Based Simultaneous PH and Nitrite Colorimetric Determination for Paper Microfluidic Devices. *Anal. Chem.* **2014**, *86*, 9554–9562. https://doi.org/10.1021/ac5019205.
68. Mazurkiewicz, W.; Podrażka, M.; Jarosińska, E.; Kappalakandy Valapil, K.; Wiloch, M.; Jönsson-Niedziółka, M.; Witkowska Nery, E. Paper-Based Electrochemical Sensors and How to Make Them (Work). *ChemElectroChem* **2020**, *7*, 2939–2956. https://doi.org/10.1002/celc.202000512.
69. Shen, L.-L.; Zhang, G.-R.; Etzold, B.J.M. Paper-Based Microfluidics for Electrochemical Applications. *ChemElectroChem* **2020**, *7*, 10–30. https://doi.org/10.1002/celc.201901495.
70. Sun, Y.; Jiang, Q.-Y.; Chen, F.; Cao, Y. Paper-Based Electrochemical Sensor. *Electrochem. Sci. Adv.* **2022**, *2*, e2100057. https://doi.org/10.1002/elsa.202100057.
71. Ataide, V.N.; Mendes, L.F.; Gama, L.I.L.M.; de Araujo, W.R.; Paixão, T.R.L.C. Electrochemical Paper-Based Analytical Devices: Ten Years of Development. *Anal. Methods* **2020**, *12*, 1030–1054. https://doi.org/10.1039/C9AY02350J.
72. Dungchai, W.; Chailapakul, O.; Henry, C.S. Electrochemical Detection for Paper-Based Microfluidics. *Anal. Chem.* **2009**, *81*, 5821–5826. https://doi.org/10.1021/ac9007573.
73. Cinti, S.; Moscone, D.; Arduini, F. Preparation of Paper-Based Devices for Reagentless Electrochemical (Bio)Sensor Strips. *Nat .Protoc.* **2019**, *14*, 2437–2451. https://doi.org/10.1038/s41596-019-0186-y.
74. Kuswandi, B.; Hidayat, M.A.; Noviana, E. Paper-Based Electrochemical Biosensors for Food Safety Analysis. *Biosensors* **2022**, *12*, 1088. https://doi.org/10.3390/bios12121088.
75. Dossi, N.; Toniolo, R.; Pizzariello, A.; Impellizzieri, F.; Piccin, E.; Bontempelli, G. Pencil-Drawn Paper Supported Electrodes as Simple Electrochemical Detectors for Paper-Based Fluidic Devices. *Electrophoresis* **2013**, *34*, 2085–2091. https://doi.org/10.1002/elps.201200425.
76. Ameku, W.A.; De Araujo, W.R.; Rangel, C.J.; Ando, R.A.; Paixão, T.R.L.C. Gold Nanoparticle Paper-Based Dual-Detection

Device for Forensics Applications. *ACS Appl. Nano Mater.* **2019**, *2*, 5460–5468. https://doi.org/10.1021/acsanm.9b01057.

77. Dias, A.A.; Cardoso, T.M.G.; Chagas, C.L.S.; Oliveira, V.X.G.; Munoz, R.A.A.; Henry, C.S.; Santana, M.H.P.; Paixão, T.R.L.C.; Coltro, W.K.T. Detection of Analgesics and Sedation Drugs in Whiskey Using Electrochemical Paper-Based Analytical Devices. *Electroanalysis* **2018**, *30*, 2250–2257. https://doi.org/10.1002/elan.201800308.
78. Rocha, D.S.; Duarte, L.C.; Silva-Neto, H.A.; Chagas, C.L.S.; Santana, M.H.P.; Antoniosi Filho, N.R.; Coltro, W.K.T. Sandpaper-Based Electrochemical Devices Assembled on a Reusable 3D-Printed Holder to Detect Date Rape Drug in Beverages. *Talanta* **2021**, *232*, 122408. https://doi.org/10.1016/j.talanta.2021.122408.
79. Dossi, N.; Terzi, F.; Piccin, E.; Toniolo, R.; Bontempelli, G. Rapid Prototyping of Sensors and Conductive Elements by Day-to-Day Writing Tools and Emerging Manufacturing Technologies. *Electroanalysis* **2016**, *28*, 250–264. https://doi.org/10.1002/elan.201500361.
80. Dossi, N.; Toniolo, R.; Impellizzieri, F.; Bontempelli, G. Doped Pencil Leads for Drawing Modified Electrodes on Paper-Based Electrochemical Devices. *J. Electroanal. Chem.* **2014**, *722*, 90–94. https://doi.org/10.1016/j.jelechem.2014.03.038.
81. Jangid, A.R.; Strong, E.B.; Chuang, J.; Martinez, A.W.; Martinez, N.W. Evaluation of Commercially-Available Conductive Filaments for 3D Printing Flexible Circuits on Paper. *PeerJ Mater. Sci.* **2022**, *4*, e21. https://doi.org/10.7717/peerj-matsci.21.
82. Narang, J.; Malhotra, N.; Singhal, C.; Mathur, A.; Krishna, A.K.; Pundir, C.S. Detection of Alprazolam with a Lab on Paper Economical Device Integrated with Urchin like Ag@ Pd Shell Nano-Hybrids. *Mater. Sci. Eng. C* **2017**, *80*, 728–735. https://doi.org/10.1016/j.msec.2016.11.128.
83. Narang, J.; Singhal, C.; Mathur, A.; Khanuja, M.; Varshney, A.; Garg, K.; Dahiya, T.; Pundir, C.S. Lab on Paper Chip Integrated with Si@GNRs for Electroanalysis of Diazepam. *Anal. Chim. Acta* **2017**, *980*, 50–57. https://doi.org/10.1016/j.aca.2017.05.006.
84. Saisahas, K.; Soleh, A.; Promsuwan, K.; Saichanapan, J.; Phonchai, A.; Sadiq, N.S.M.; Teoh, W.K.; Chang, K.H.; Abdullah, A.F.L.; Limbut, W. Nanocoral-like Polyaniline-Modified Graphene-Based Electrochemical Paper-Based Analytical Device for a Portable Electrochemical Sensor for Xylazine Detection. *ACS Omega* **2022**, *7*, 13913–13924. https://doi.org/10.1021/acsomega.2c00295.
85. Narang, J.; Malhotra, N.; Singhal, C.; Mathur, A.; Chakraborty, D.; Anil, A.; Ingle, A.; Pundir, C.S. Point of Care with Micro Fluidic Paper Based Device Integrated with Nano Zeolite–Graphene Oxide Nanoflakes for Electrochemical Sensing of Ketamine. *Biosens. Bioelectron.* **2017**, *88*, 249–257. https://doi.org/10.1016/j.bios.2016.08.043.
86. Pholsiri, T.; Khamcharoen, W.; Vimolmangkang, S.; Siangproh, W.; Chailapakul, O. Paper-Based Electrochemical Sensor for Simultaneous Detection of Salivary Δ^9-Tetrahydrocannabinol and Thiocyanate to Differentiate Illegal Cannabis Smokers. *Sens. Actuators B Chem.* **2023**, *383*, 133571. https://doi.org/10.1016/j.snb.2023.133571.
87. Pang, R.; Zhu, Q.; Wei, J.; Meng, X.; Wang, Z. Enhancement of the Detection Performance of Paper-Based Analytical Devices by Nanomaterials. *Molecules* **2022**, *27*, 508. https://doi.org/10.3390/molecules27020508.
88. Holman, J.B.; Shi, Z.; Fadahunsi, A.A.; Li, C.; Ding, W. Advances on Microfluidic Paper-Based Electroanalytical Devices. *Biotechnol. Adv.* **2023**, *63*, 108093. https://doi.org/10.1016/j.biotechadv.2022.108093.
89. Liu, X.; Gao, X.; Yang, L.; Zhao, Y.; Li, F. Metal-Organic Framework-Functionalized Paper-Based Electrochemical Biosensor for Ultrasensitive Exosome Assay. *Anal. Chem.* **2021**, *93*, 11792–11799. https://doi.org/10.1021/acs.analchem.1c02286.
90. McNeill, L.; Pearson, C.; Megson, D.; Norrey, J.; Watson, D.; Ashworth, D.; Linton, P.E.; Sutcliffe, O.B.; Shaw, K.J. Origami Chips: Development and Validation of a Paper-Based Lab-on-a-Chip Device for the Rapid and Cost-Effective Detection of 4-Methylmethcathinone (Mephedrone) and Its Metabolite, 4-Methylephedrine in Urine. *Forensic Chem.* **2021**, *22*, 100293. https://doi.org/10.1016/j.forc.2020.100293.
91. Oh, J.M.; Chow, K.F. Recent Developments in Electrochemical Paper-Based Analytical Devices. *Anal. Methods* **2015**, *7*, 7951–7960. https://doi.org/10.1039/c5ay01724f.
92. Renault, C.; Anderson, M.J.; Crooks, R.M. Electrochemistry in Hollow-Channel Paper Analytical Devices. *J. Am. Chem. Soc.* **2014**, *136*, 4616–4623. https://doi.org/10.1021/ja4118544.
93. Marques, A.C.; Pinheiro, T.; Martins, G.V.; Cardoso, A.R.; Martins, R.; Sales, M.G.; Fortunato, E. Non-Enzymatic Lab-on-Paper Devices for Biosensing Applications. *Compr. Anal. Chem.* **2020**, *89*, 189–237. https://doi.org/10.1016/bs.coac.2020.05.001.
94. Abd-Rabboh, H.S.M.; El-Galil, E.; Amr, A.; Elsayed, E.A.; Sayed, A.Y.A.; Kamel, A.H. Paper-Based Potentiometric Sensing Devices Modified with Chemically Reduced Graphene Oxide (CRGO) for Trace Level Determination of Pholcodine (Opiate Derivative Drug). *RSC Adv.* **2021**, *11*, 12227–12234. https://doi.org/10.1039/D1RA00581B.
95. Özbek, O.; Berkel, C. Recent Advances in Potentiometric Analysis: Paper–Based Devices. *Sens. Int.* **2022**, *3*, 100189. https://doi.org/10.1016/j.sintl.2022.100189.
96. Tantawy, M.A.; Mohamed, E.H.; Yehia, A.M. All Solid-State Miniaturized Potentiometric Sensors for Flunitrazepam Determination in Beverages. *Microchim. Acta* **2021**, *188*, 192. https://doi.org/10.1007/s00604-021-04851-9.
97. Arantes, I.V.S.; Paixão, T.R.L.C. Couple Batch-Injection Analysis and Microfluidic Paper-Based Analytical Device: A Simple and Disposable Alternative to Conventional BIA Apparatus. *Talanta* **2022**, *240*, 123201. https://doi.org/10.1016/j.talanta.2021.123201.
98. Pholsiri, T.; Lomae, A.; Pungjunun, K.; Vimolmangkang, S.; Siangproh, W.; Chailapakul, O. A Chromatographic Paper-Based Electrochemical Device to Determine Δ^9-Tetrahydrocannabinol and Cannabidiol in Cannabis Oil. *Sens. Actuators B Chem.* **2022**, *355*, 131353. https://doi.org/10.1016/j.snb.2021.131353.
99. Lan, W.-J.; Maxwell, E.J.; Parolo, C.; Bwambok, D.K.; Subramaniam, A.B.; Whitesides, G.M. Paper-Based Electroanalytical Devices with an Integrated, Stable Reference Electrode. *Lab Chip* **2013**, *13*, 4103. https://doi.org/10.1039/c3lc50771h.
100. Narang, J.; Singhal, C.; Khanuja, M.; Mathur, A.; Jain, A.; Pundir, C.S. Hydrothermally Synthesized Zinc Oxide Nanorods

Incorporated on Lab-on-Paper Device for Electrochemical Detection of Recreational Drug. *Artif. Cells Nanomed. Biotechnol.* **2017**, *46*, 1–8. https://doi.org/10.1080/21691401.2017.1381614.
101. Fava, E.L.; Martimiano do Prado, T.; Almeida Silva, T.; Cruz de Moraes, F.; Censi Faria, R.; Fatibello-Filho, O. New Disposable Electrochemical Paper-Based Microfluidic Device with Multiplexed Electrodes for Biomarkers Determination in Urine Sample. *Electroanalysis* **2020**, *32*, 1075–1083. https://doi.org/10.1002/elan.201900641.
102. Dossi, N.; Toniolo, R.; Impellizzieri, F.; Tubaro, F.; Bontempelli, G.; Terzi, F.; Piccin, E. A Paper-Based Platform with a Pencil-Drawn Dual Amperometric Detector for the Rapid Quantification of Ortho-Diphenols in Extravirgin Olive Oil. *Anal. Chim. Acta* **2017**, *950*, 41–48. https://doi.org/10.1016/j.aca.2016.11.030.
103. Dossi, N.; Toniolo, R.; Terzi, F.; Sdrigotti, N.; Tubaro, F.; Bontempelli, G. A Cotton Thread Fluidic Device with a Wall-Jet Pencil-Drawn Paper Based Dual Electrode Detector. *Anal. Chim. Acta* **2018**, *1040*, 74–80. https://doi.org/10.1016/j.aca.2018.06.061.
104. Yamamoto, S.; Uno, S. Redox Cycling Realized in Paper-Based Biochemical Sensor for Selective Detection of Reversible Redox Molecules Without Micro/Nano Fabrication Process. *Sensors* **2018**, *18*, 730. https://doi.org/10.3390/s18030730.
105. Noviana, E.; Klunder, K.J.; Channon, R.B.; Henry, C.S. Thermoplastic Electrode Arrays in Electrochemical Paper-Based Analytical Devices. *Anal. Chem.* **2019**, *91*, 2431–2438. https://doi.org/10.1021/acs.analchem.8b05218.
106. Santhiago, M.; Wydallis, J.B.; Kubota, L.T.; Henry, C.S. Construction and Electrochemical Characterization of Microelectrodes for Improved Sensitivity in Paper-Based Analytical Devices. *Anal. Chem.* **2013**, *85*, 5233–5239. https://doi.org/10.1021/ac400728y.
107. Langer, J.; Jimenez de Aberasturi, D.; Aizpurua, J.; Alvarez-Puebla, R.A.; Auguié, B.; Baumberg, J.J.; Bazan, G.C.; Bell, S.E.J.; Boisen, A.; Brolo, A.G.; et al. Present and Future of Surface-Enhanced Raman Scattering. *ACS Nano* **2020**, *14*, 28–117. https://doi.org/10.1021/acsnano.9b04224.
108. Le Ru, E.C.; Etchegoin, P.G. A Quick Overview of Surface-Enhanced Raman Spectroscopy. In *Principles of Surface-Enhanced Raman Spectroscopy*; Elsevier: Amsterdam, The Netherlands, 2009; pp. 1–27. ISBN 978-0-444-52779-0.
109. Bonifacio, A. Nanostrucured Substrates for Surface-Enhanced Raman Scattering Spectroscopy. In *Nanomaterials for Theranostics and Tissue Engineering*; Elsevier: Amsterdam, The Netherlands, 2020; pp. 137–174. ISBN 978-0-12-817838-6.
110. Betz, J.F.; Yu, W.W.; Cheng, Y.; White, I.M.; Rubloff, G.W. Simple SERS Substrates: Powerful, Portable, and Full of Potential. *Phys. Chem. Chem. Phys.* **2014**, *16*, 2224–2239. https://doi.org/10.1039/C3CP53560F.
111. Vicente, A.T.; Araújo, A.; Mendes, M.J.; Nunes, D.; Oliveira, M.J.; Sanchez-Sobrado, O.; Ferreira, M.P.; Águas, H.; Fortunato, E.; Martins, R. Multifunctional Cellulose-Paper for Light Harvesting and Smart Sensing Applications. *J. Mater. Chem. C* **2018**, *6*, 3143–3181. https://doi.org/10.1039/C7TC05271E.
112. Pilot, R.; Signorini, R.; Durante, C.; Orian, L.; Bhamidipati, M.; Fabris, L. A Review on Surface-Enhanced Raman Scattering. *Biosensors* **2019**, *9*, 57. https://doi.org/10.3390/bios9020057.
113. Ogundare, S.A.; van Zyl, W.E. A Review of Cellulose-Based Substrates for SERS: Fundamentals, Design Principles, Applications. *Cellulose* **2019**, *26*, 6489–6528. https://doi.org/10.1007/s10570-019-02580-0.
114. Liu, H.; He, Y.; Cao, K. Flexible Surface-Enhanced Raman Scattering Substrates: A Review on Constructions, Applications, and Challenges. *Adv. Mater. Interfaces* **2021**, *8*, 2100982. https://doi.org/10.1002/admi.202100982.
115. Yu, W.W.; White, I.M. Inkjet Printed Surface Enhanced Raman Spectroscopy Array on Cellulose Paper. *Anal. Chem.* **2010**, *82*, 9626–9630. https://doi.org/10.1021/ac102475k.
116. Tay, L.-L.; Poirier, S.; Ghaemi, A.; Hulse, J.; Wang, S. Iodide Functionalized Paper-Based SERS Sensors for Improved Detection of Narcotics. *Front. Chem.* **2021**, *9*, 680556. https://doi.org/10.3389/fchem.2021.680556.
117. Polavarapu, L.; Porta, A.L.; Novikov, S.M.; Coronado-Puchau, M.; Liz-Marzán, L.M. Pen-on-Paper Approach Toward the Design of Universal Surface Enhanced Raman Scattering Substrates. *Small* **2014**, *10*, 3065–3071. https://doi.org/10.1002/smll.201400438.
118. Oliveira, M.J.; Quaresma, P.; Peixoto de Almeida, M.; Araújo, A.; Pereira, E.; Fortunato, E.; Martins, R.; Franco, R.; Águas, H. Office Paper Decorated with Silver Nanostars—An Alternative Cost Effective Platform for Trace Analyte Detection by SERS. *Sci. Rep.* **2017**, *7*, 2480. https://doi.org/10.1038/s41598-017-02484-8.
119. Oh, K.; Lee, M.; Lee, S.G.; Jung, D.H.; Lee, H.L. Cellulose Nanofibrils Coated Paper Substrate to Detect Trace Molecules Using Surface-Enhanced Raman Scattering. *Cellulose* **2018**, *25*, 3339–3350. https://doi.org/10.1007/s10570-018-1806-3.
120. Laing, S.; Jamieson, L.E.; Faulds, K.; Graham, D. Surface-Enhanced Raman Spectroscopy for in Vivo Biosensing. *Nat. Rev. Chem.* **2017**, *1*, 0060. https://doi.org/10.1038/s41570-017-0060.
121. Goodacre, R.; Graham, D.; Faulds, K. Recent Developments in Quantitative SERS: Moving towards Absolute Quantification. *TrAC Trends Anal. Chem.* **2018**, *102*, 359–368. https://doi.org/10.1016/j.trac.2018.03.005.
122. Shen, W.; Lin, X.; Jiang, C.; Li, C.; Lin, H.; Huang, J.; Wang, S.; Liu, G.; Yan, X.; Zhong, Q.; et al. Reliable Quantitative SERS Analysis Facilitated by Core-Shell Nanoparticles with Embedded Internal Standards. *Angew. Chem.* **2015**, *54*, 7308–7312. https://doi.org/10.1002/anie.201502171.
123. Duncan, A.; Taylor, A.; Leese, E.; Allen, S.; Morton, J.; McAdam, J. Homicidal Arsenic Poisoning. *Ann. Clin. Biochem.* **2015**, *52*, 510–515. https://doi.org/10.1177/0004563214559222.
124. Dueñas-Laita, A.; Pérez-Miranda, M.; González-López, M.A.; Martín-Escudero, J.C.; Ruiz-Mambrilla, M.; Blanco-Varela, J. Acute Arsenic Poisoning. *Lancet* **2005**, *365*, 1982. https://doi.org/10.1016/S0140-6736(05)66670-6.
125. Saadati, A.; Farshchi, F.; Hasanzadeh, M.; Liu, Y.; Seidi, F. Colorimetric and Naked-Eye Detection of Arsenic(Iii) Using a Paper-Based Colorimetric Device Decorated with Silver Nanoparticles. *RSC Adv.* **2022**, *12*, 21836–21850. https://doi.org/10.1039/d2ra02820d.
126. Swezey, R.; Shinn, W.; Green, C.; Drover, D.R.; Hammer, G.B.; Schulman, S.R.; Zajicek, A.; Jett, D.A.; Boss, G.R. Comparison of

a New Cobinamide-Based Method to a Standard Laboratory Method for Measuring Cyanide in Human Blood. *J. Anal. Toxicol.* **2013**, *37*, 382–385. https://doi.org/10.1093/jat/bkt037.
127. Wachełko, O.; Chłopaś-Konowałek, A.; Zawadzki, M.; Szpot, P. Old Poison, New Problem: Cyanide Fatal Intoxications Associated with Internet Shopping. *J. Anal. Toxicol.* **2022**, *46*, e52–e59. https://doi.org/10.1093/jat/bkab039.
128. Felscher, D.; Wulfmeyer, M. A New Specific Method to Detect Cyanide in Body Fluids, Especially Whole Blood, by Fluorimetry. *J. Anal. Toxicol.* **1998**, *22*, 363–366. https://doi.org/10.1093/jat/22.5.363.
129. Petruci, J.F.D.S.; Hauser, P.C.; Cardoso, A.A. Colorimetric Paper-Based Device for Gaseous Hydrogen Cyanide Quantification Based on Absorbance Measurements. *Sens. Actuators B Chem.* **2018**, *268*, 392–397. https://doi.org/10.1016/j.snb.2018.04.101.
130. Sheini, A.; Aseman, M.D.; Bordbar, M.M. Origami Paper Analytical Assay Based on Metal Complex Sensor for Rapid Determination of Blood Cyanide Concentration in Fire Survivors. *Sci. Rep.* **2021**, *11*, 3521. https://doi.org/10.1038/s41598-021-83186-0.
131. Wirojsaengthong, S.; Aryuwananon, D.; Aeungmaitrepirom, W.; Pulpoka, B.; Tuntulani, T. A Colorimetric Paper-Based Optode Sensor for Highly Sensitive and Selective Determination of Thiocyanate in Urine Sample Using Cobalt Porphyrin Derivative. *Talanta* **2021**, *231*, 122371. https://doi.org/10.1016/j.talanta.2021.122371.
132. Thepchuay, Y.; Sonsa-Ard, T.; Ratanawimarnwong, N.; Auparakkitanon, S.; Sitanurak, J.; Nacapricha, D. Paper-Based Colorimetric Biosensor of Blood Alcohol with in-Situ Headspace Separation of Ethanol from Whole Blood. *Anal. Chim. Acta* **2020**, *1103*, 115–121. https://doi.org/10.1016/j.aca.2019.12.043.
133. Hikin, L.J.; Ho, J.; Morley, S.R.; Ahluwalia, A.; Smith, P.R. Sodium Nitrite Poisoning: A Series of 20 Fatalities in Which Post-Mortem Blood Nitrite and Nitrate Concentrations Are Reported. *Forensic Sci. Int.* **2023**, *345*, 111610. https://doi.org/10.1016/j.forsciint.2023.111610.
134. Zhang, X.-X.; Song, Y.-Z.; Fang, F.; Wu, Z.-Y. Sensitive Paper-Based Analytical Device for Fast Colorimetric Detection of Nitrite with Smartphone. *Anal. Bioanal. Chem.* **2018**, *410*, 2665–2669. https://doi.org/10.1007/s00216-018-0965-2.
135. Yu, P.; Deng, M.; Yang, Y. New Single-Layered Paper-Based Microfluidic Devices for the Analysis of Nitrite and Glucose Built via Deposition of Adhesive Tape. *Sensors* **2019**, *19*, 4082. https://doi.org/10.3390/s19194082.
136. Yu, P.; Deng, M.; Yang, Y.; Nie, B.; Zhao, S. 3d Microfluidic Devices in a Single Piece of Paper for the Simultaneous Determination of Nitrite and Thiocyanate. *Sensors* **2020**, *20*, 4118. https://doi.org/10.3390/s20154118.
137. de Oliveira, R.A.G.; Camargo, F.; Pesquero, N.C.; Faria, R.C. A Simple Method to Produce 2D and 3D Microfluidic Paper-Based Analytical Devices for Clinical Analysis. *Anal. Chim. Acta* **2017**, *957*, 40–46. https://doi.org/10.1016/j.aca.2017.01.002.
138. Klasner, S.A.; Price, A.K.; Hoeman, K.W.; Wilson, R.S.; Bell, K.J.; Culbertson, C.T. Paper-Based Microfluidic Devices for Analysis of Clinically Relevant Analytes Present in Urine and Saliva. *Anal. Bioanal. Chem.* **2010**, *397*, 1821–1829. https://doi.org/10.1007/s00216-010-3718-4.
139. Bhakta, S.A.; Borba, R.; Taba, M.; Garcia, C.D.; Carrilho, E. Determination of Nitrite in Saliva Using Microfluidic Paper-Based Analytical Devices. *Anal. Chim. Acta* **2014**, *809*, 117–122. https://doi.org/10.1016/j.aca.2013.11.044.
140. Ferreira, F.T.S.M.; Mesquita, R.B.R.; Rangel, A.O.S.S. Novel Microfluidic Paper-Based Analytical Devices (MPADs) for the Determination of Nitrate and Nitrite in Human Saliva. *Talanta* **2020**, *219*, 121183. https://doi.org/10.1016/j.talanta.2020.121183.
141. Cardoso, T.M.G.; Garcia, P.T.; Coltro, W.K.T. Colorimetric Determination of Nitrite in Clinical, Food and Environmental Samples Using Microfluidic Devices Stamped in Paper Platforms. *Anal. Methods* **2015**, *7*, 7311–7317. https://doi.org/10.1039/c5ay00466g.
142. Mollaie, E.; Asiaei, S.; Aryan, H. Nitrite Enhanced Detection from Saliva by Simple Geometrical Modifications of Paper-Based Micromixers. *Microfluid. Nanofluidics* **2022**, *26*, 88. https://doi.org/10.1007/s10404-022-02596-2.
143. Hou, Y.; Guo, Y.; Ma, X.; Lv, C.; Yang, M.; Yao, S.; Jin, Y.; Li, B.; Liu, W. Ring-Oven-Assisted In Situ Synthesis of Metal–Organic Frameworks on the Lab-On-Paper Device for Chemiluminescence Detection of Nitrite in Whole Blood. *Anal. Chem.* **2023**, *95*, 4362–4370. https://doi.org/10.1021/acs.analchem.2c04765.
144. Ansari, N.; Lodha, A.; Pandya, A.; Menon, S.K. Determination of Cause of Death Using Paper-Based Microfluidic Device as a Colorimetric Probe. *Anal. Methods* **2017**, *9*, 5632–5639. https://doi.org/10.1039/C7AY01784G.
145. Wei, X.; Tian, T.; Jia, S.; Zhu, Z.; Ma, Y.; Sun, J.; Lin, Z.; Yang, C.J. Target-Responsive DNA Hydrogel Mediated "Stop-Flow" Microfluidic Paper-Based Analytic Device for Rapid, Portable and Visual Detection of Multiple Targets. *Anal. Chem.* **2015**, *87*, 4275–4282. https://doi.org/10.1021/acs.analchem.5b00532.
146. Tian, T.; Wei, X.; Jia, S.; Zhang, R.; Li, J.; Zhu, Z.; Zhang, H.; Ma, Y.; Lin, Z.; Yang, C.J. Integration of Target Responsive Hydrogel with Cascaded Enzymatic Reactions and Microfluidic Paper-Based Analytic Devices (MPADs) for Point-of-Care Testing (POCT). *Biosens. Bioelectron.* **2016**, *77*, 537–542. https://doi.org/10.1016/j.bios.2015.09.049.
147. He, M.; Li, Z.; Ge, Y.; Liu, Z. Portable Upconversion Nanoparticles-Based Paper Device for Field Testing of Drug Abuse. *Anal. Chem.* **2016**, *88*, 1530–1534. https://doi.org/10.1021/acs.analchem.5b04863.
148. Yu, W.W.; White, I.M. Inkjet-Printed Paper-Based SERS Dipsticks and Swabs for Trace Chemical Detection. *Analyst* **2013**, *138*, 1020–1025. https://doi.org/10.1039/C2AN36116G.
149. Burr, D.S.; Fatigante, W.L.; Lartey, J.A.; Jang, W.; Stelmack, A.R.; McClurg, N.W.; Standard, J.M.; Wieland, J.R.; Kim, J.-H.; Mulligan, C.C.; et al. Integrating SERS and PSI-MS with Dual Purpose Plasmonic Paper Substrates for On-Site Illicit Drug Confirmation. *Anal. Chem.* **2020**, *92*, 6676–6683. https://doi.org/10.1021/acs.analchem.0c00562.
150. Han, S.; Zhang, C.; Lin, S.; Sha, X.; Hasi, W. Sensitive and Reliable Identification of Fentanyl Citrate in Urine and Serum Using Chloride Ion-Treated Paper-Based SERS Substrate. *Spectrochim. Acta Part A Mol. Biomol. Spectrosc.* **2021**, *251*, 119463.

https://doi.org/10.1016/j.saa.2021.119463.
151. Haddad, A.; Comanescu, M.A.; Green, O.; Kubic, T.A.; Lombardi, J.R. Detection and Quantitation of Trace Fentanyl in Heroin by Surface-Enhanced Raman Spectroscopy. *Anal. Chem.* **2018**, *90*, 12678–12685. https://doi.org/10.1021/acs.analchem.8b02909.
152. Tay, L.-L.; Poirier, S.; Ghaemi, A.; Hulse, J. Inkjet-Printed Paper-Based Surface Enhanced Raman Scattering (SERS) Sensors for the Detection of Narcotics. *MRS Adv.* **2022**, *7*, 190–196. https://doi.org/10.1557/s43580-022-00257-8.
153. Chen, C.-A.; Wang, P.-W.; Yen, Y.-C.; Lin, H.-L.; Fan, Y.-C.; Wu, S.-M.; Chen, C.-F. Fast Analysis of Ketamine Using a Colorimetric Immunosorbent Assay on a Paper-Based Analytical Device. *Sens. Actuators B Chem.* **2019**, *282*, 251–258. https://doi.org/10.1016/j.snb.2018.11.071.
154. Vieira, O.; Moal, A.; Milan, N.; Sibille, P.; Deffontaine, G.; Ghysel-Laporte, M.-H. Paradoxal Use of Stimulants in Drug-Facilitated Crime. *Toxicol. Anal. Et Clin.* **2014**, *26*, S39. https://doi.org/10.1016/S2352-0078(14)70082-4.
155. Teerinen, T.; Lappalainen, T.; Erho, T. A Paper-Based Lateral Flow Assay for Morphine. *Anal. Bioanal. Chem.* **2014**, *406*, 5955–5965. https://doi.org/10.1007/s00216-014-8001-7.
156. Food and Drug Administration (FDA). Bioanalytical Method Validation Guidance for Industry. 2018. Available online: (accessed on).
157. Moulahoum, H.; Ghorbanizamani, F.; Timur, S. Paper-Based Lateral Flow Assay Using Rhodamine B–Loaded Polymersomes for the Colorimetric Determination of Synthetic Cannabinoids in Saliva. *Microchim. Acta* **2021**, *188*, 1–12. https://doi.org/10.1007/s00604-021-05062-y.
158. Son, S.U.; Jang, S.; Kang, B.; Kim, J.; Lim, J.; Seo, S.; Kang, T.; Jung, J.; Lee, K.-S.; Kim, H.; et al. Colorimetric Paper Sensor for Visual Detection of Date-Rape Drug γ-Hydroxybutyric Acid (GHB). *Sens. Actuators B Chem.* **2021**, *347*, 130598. https://doi.org/10.1016/j.snb.2021.130598.
159. Yehia, A.M.; Farag, M.A.; Tantawy, M.A. A Novel Trimodal System on a Paper-Based Microfluidic Device for on-Site Detection of the Date Rape Drug "Ketamine." *Anal. Chim. Acta* **2020**, *1104*, 95–104. https://doi.org/10.1016/j.aca.2020.01.002.
160. Melchior, S.E.; Nielsen, M.K.K.; Oropeza, A.R.; Banner, J.; Johansen, S.S. Detection of Scopolamine in Urine and Hair in a Drug-Facilitated Sexual Assault. *Forensic Sci. Int.* **2023**, *347*, 111678. https://doi.org/10.1016/j.forsciint.2023.111678.
161. Dias, B.C.; Batista, A.D.; da Silveira Petruci, J.F. MOPTO: A Microfluidic Paper-Based Optoelectronic Tongue as Presumptive Tests for the Discrimination of Alkaloid Drugs for Forensic Purposes. *Anal. Chim. Acta* **2021**, *1187*, 339141. https://doi.org/10.1016/j.aca.2021.339141.
162. Mustafa, F.; Carhart, M.; Andreescu, S. A 3D-Printed Breath Analyzer Incorporating CeO_2 Nanoparticles for Colorimetric Enzyme-Based Ethanol Sensing. *ACS Appl. Nano Mater.* **2021**, *4*, 9361–9369. https://doi.org/10.1021/acsanm.1c01826.
163. Alder, R.; Hong, J.; Chow, E.; Fang, J.; Isa, F.; Ashford, B.; Comte, C.; Bendavid, A.; Xiao, L.; Ostrikov, K.; et al. Application of Plasma-Printed Paper-Based SERS Substrate for Cocaine Detection. *Sensors* **2021**, *21*, 810. https://doi.org/10.3390/s21030810.
164. Ameku, W.A.; Gonçalves, J.M.; Ataide, V.N.; Ferreira Santos, M.S.; Gutz, I.G.R.; Araki, K.; Paixão, T.R.L.C. Combined Colorimetric and Electrochemical Measurement Paper-Based Device for Chemometric Proof-of-Concept Analysis of Cocaine Samples. *ACS Omega* **2021**, *6*, 594–605. https://doi.org/10.1021/acsomega.0c05077.
165. Fedick, P.W.; Pu, F.; Morato, N.M.; Cooks, R.G. Identification and Confirmation of Fentanyls on Paper Using Portable Surface Enhanced Raman Spectroscopy and Paper Spray Ionization Mass Spectrometry. *J. Am. Soc. Mass Spectrom.* **2020**, *31*, 735–741. https://doi.org/10.1021/jasms.0c00004.
166. NASA Technology Readiness Levels Demystified. Available online: http://www.nasa.gov/topics/aeronautics/features/trl_demystified.html (accessed on 12 June 2023).
167. Kunkel, H.G.; Tiselius, A. Electrophoresis of Proteins on Filter Paper. *J. Gen. Physiol.* **1951**, *35*, 89–118.
168. Silva-Neto, H.A.; Arantes, I.V.S.; Ferreira, A.L.; do Nascimento, G.H.M.; Meloni, G.N.; de Araujo, W.R.; Paixão, T.R.L.C.; Coltro, W.K.T. Recent Advances on Paper-Based Microfluidic Devices for Bioanalysis. *TrAC Trends Anal. Chem.* **2023**, *158*, 116893. https://doi.org/10.1016/j.trac.2022.116893.
169. Li, T.; Liang, B.; Ye, Z.; Zhang, L.; Xu, S.; Tu, T.; Zhang, Y.; Cai, Y.; Zhang, B.; Fang, L.; et al. An Integrated and Conductive Hydrogel-Paper Patch for Simultaneous Sensing of Chemical–Electrophysiological Signals. *Biosens. Bioelectron.* **2022**, *198*, 113855. https://doi.org/10.1016/j.bios.2021.113855.
170. Xu, Y.; Fei, Q.; Page, M.; Zhao, G.; Ling, Y.; Stoll, S.B.; Yan, Z. Paper-Based Wearable Electronics. *iScience* **2021**, *24*, 102736. https://doi.org/10.1016/j.isci.2021.102736.
171. Criscuolo, F.; Cantu, F.; Taurino, I.; Carrara, S.; De Micheli, G. A Wearable Electrochemical Sensing System for Non-Invasive Monitoring of Lithium Drug in Bipolar Disorder. *IEEE Sens. J.* **2021**, *21*, 9649–9656. https://doi.org/10.1109/JSEN.2020.3009538.
